# Impact of the Ceria Particle Oxidation State on the Collecting Properties of Sophorolipids and Benzohydroxamic Acid

**DOI:** 10.1021/acsomega.4c04818

**Published:** 2024-09-27

**Authors:** Vladislav Slabov, Hanumantha Rao Kota, Irina V. Chernyshova

**Affiliations:** † Department of Geoscience and Petroleum, Norwegian University of Science and Technology (NTNU), Trondheim NO-7031, Norway; ‡ Department of Earth and Environmental Engineering, Columbia University, New York, New York 10027, United States

## Abstract

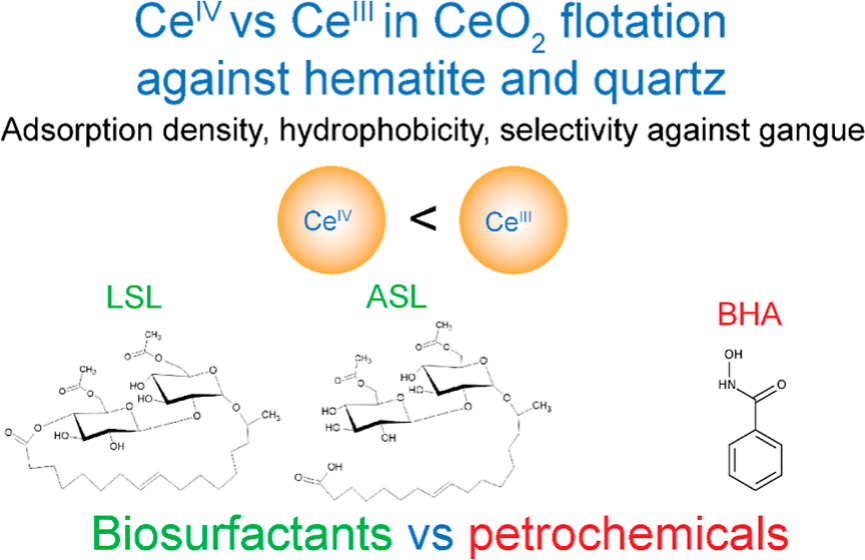

The search for environmentally friendly alternatives to petroleum-based reagents in mineral processing requires fundamental studies of novel chemicals in model mineral systems. In this study, we evaluate the potential of acidic (ASL) and lactonic sophorolipids (LSL) as collectors in the froth flotation of ultrafine ceria, which serves as a model rare earth mineral (REM). We compare these two biosurfactants to a conventional petroleum-based collector, benzohydroxamic acid (BHA), in the flotation of ceria against hematite and quartz particles. Our research shows the effect of the oxidation state of ceria on its interaction with both conventional and sophorolipid collectors, which can serve as a tool for selective separation in the applied chemistry of froth flotation. It was found that the affinity of the metal oxides to the biosurfactants at pH 4 decreases in the order of α-Fe_2_O_3_ > CeO_2_^(red)^ > CeO_2_^(ox)^, where the best collector of ultrafine ceria against hematite is BHA. To support our findings, we study collector–mineral interactions through mini-flotation tests, adsorption density, contact angle, and zeta potential analyses. In addition, we evaluate the stability of the froth during the flotation of the biosurfactants. Our results indicate that modifying the oxidation state of ceria and using sophorolipids hold promise for the sustainable flotation of REM.

## 1 Introduction

The growing demand for rare earth elements (REEs) to feed the green energy infrastructure cannot be met without the adoption of more sustainable practices for the extraction of rare earth minerals (REMs) from both primary (ore) and secondary (waste) sources.^1,2^ One of the main industrial technologies for the extracting REM from both ores and REM-rich tailings is froth flotation.^3−5^ It employs the selective adsorption of surfactants (amphoteric ligands, which are called flotation collectors) onto target mineral particles to make them hydrophobic. This allows them to be separated by bubbles and gravity. However, REMs are typically highly dispersed in ores.^6^ Hence, to liberate them, ores are ground to ultrafine sizes (<20–30 μm), creating the so-called problem of fines.^7,8^ Specifically, a high fraction of ultrafine particles affects flotation selectivity and leads to unwanted froth stabilization.^5,8−12^ The problem of fines leads to an abundance of fine and ultrafine particles in the tailings (flotation waste), which makes the tailings difficult to reprocess.^4^ For example, REE-containing tailings from a South Australian mining operation, which are rich in iron oxides and silicates, contain approximately 67% of paramagnetic iron oxide particles smaller than 38 μm.^13^ Another notable challenge in the flotation of REM is the similarity of their surface chemistry to that of the metal oxide and carbonate gangue commonly found in the fine fraction.^14−16^ Therefore, there is a need for improved flotation collectors for the more efficient extraction of ultrafine REMs.

The most common collectors of REM are fatty acids and hydroxamic acids (hydroxamate).^3,17^ Even though fatty acids meet the green chemistry criteria, they are less selective than hydroxamic acids.^3,17^ The latter form 5-membered chelating complexes with REE that are 7 orders of magnitude more stable than the 4-membered complexes of carboxylates.^18^ Unlike environmentally friendly fatty acids, hydroxamic acids are petroleum-based and toxic to the environment.^19^ Therefore, environmental sustainability goals motivate the search for more efficient collectors of fine REM among green (eco-friendly) reagents.

A promising platform for the development of green collectors is microbial biosurfactants.^20,21^ In addition to being biodegradable and low or nontoxic, they are produced by benign microbes from renewable biomass including food waste.^22−24^ Their potential as collectors is suggested by their ability to selectively coordinate metal cations, which is used in environmental remediation.^25−27^

**Figure 1:**
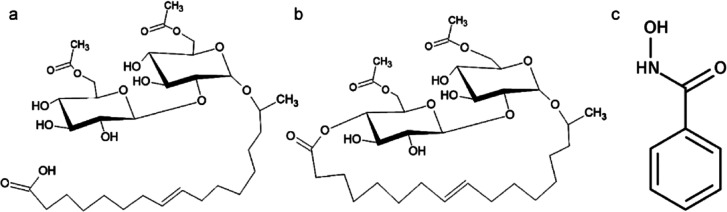
Scheme of (a) diacetylated ASL and (b) diacetylated LSL and (c) BHA. In the sophorolipids, an acetylated sophorose moiety is attached to a C18:1 hydrocarbon chain.

However, knowledge of the collecting properties of microbial biosurfactants is still in its initial stages. Acidic sophorolipid (ASL) and lactonic sophorolipid (LSL) (Figure 1b) can selectively float ultrafine copper and iron oxides against ultrafine quartz from their synthetic mixtures.^28^ Deacetylated ASL has been shown to be effective in the flotation of copper sulfide ores at bench scale.^29^ The mechanism underlying its collecting properties involves surface precipitation of six-membered chelating complexes of ASL and Cu(II).^30^ This mechanism is similar to that proposed for hydroxamic acids and REM.^31^ In particular, there is currently a lack of information about the collecting properties of biosurfactants in REM flotation.

The development of collectors for REM flotation has focused on molecules that coordinate with trivalent REEs, which represent the conventional oxidation state of REEs in REM. In particular, cerium (Ce), terbium (Tb), and praseodymium (Pr) can be oxidized to the tetravalent state, while samarium (Sm), europium (Eu), and ytterbium (Yb) can be reduced to the divalent state. However, to our knowledge, the oxidation state has not been used as a means to control the flotation process of REM containing redox-active REEs.

Thus, the objective of this study is twofold: first, to elucidate the effect of the oxidation state of ultrafine ceria (CeO_2_) particles (with sizes of −20 μm) on their flotation against ultrafine hematite (α-Fe_2_O_3_) and quartz (SiO_2_), employing diacetylated ASL, diacetylated LSL, and benzohydroxamic acid (BHA) as collectors (Figure 1). Second, we compare the collecting properties of the sophorolipids and BHA in the flotation of the ultrafine minerals. Through these objectives, this study will improve our understanding of the redox properties of oxides in flotation processes and expand our knowledge of sophorolipid biosurfactants as mineral collectors.

Cerium is the most abundant representative of the redox active REE. The main REMs are bastnäsite (REE)CO_3_F, monazite (REE)PO_4_, and (REE,Y)PO_4_ xenotime, as well as lateritic ion-adsorption clays, which contain 45–50% of Ce.^32,33^ Even though Ce is mostly trivalent in REM, it can be oxidized during ore storage, given that the bastnäsite surface contains a significant admixture of Ce^IV^.^34^ Cerianite (CeO_2_) has been found in lateritic soils. Cerium in red mud also occurs predominantly as Ce^IV^.^35^ The Ce^IV^ ions on the CeO_2_ surface are easily and reversibly reduced to Ce^III^, which is accompanied by the reversible formation of O-vacancies.^36^ Ce^III^ cations and O-vacancies form frustrated Lewis pairs—Lewis acids and bases that are sterically hindered in forming Lewis acid-base adducts. Frustrated Lewis pairs are responsible for the unusual catalytic chemistry of ceria, which includes the catalytic decomposition of adsorbed organic molecules.^36,37^ Therefore, the interaction of oxidized ceria with collectors is expected to involve redox reactions.

The effect of the cerium oxidation state on the separation process is limited by hydrometallurgical application. However, in an alkaline environment, Ce(OH)_3_ precipitates and can be further oxidized to form the [Ce(OH)_*x*_(H_2_O)_*y*_]^(4–x)+^ complex.^38^ Lee, Bogart, Carroll, and Schelter^39^ suggested that complexation of *N*-phenyl–pivalohydroxamic acid with Ce^3+^ favors binding and oxidation of cerium on bastnasite ore surfaces. The authors suggested that the redox chemistry of cerium should be considered in REE ore beneficiation processes.

To elucidate the effect of the oxidation state of ceria flotation, we use reduced and oxidized ceria particles, denoted CeO_2_^(red)^ and CeO_2_^(ox)^, respectively. Hematite and quartz are chosen as representative gangue minerals because of their prevalence in various geological REE ores, REE-containing tailings, and red mud.^13,17,35^ The choice of hematite is also explained by its weak magnetic properties, which require froth flotation for its separation. Quartz is commonly associated with hematite. Iron oxides and silicates are typically depressed in REM flotation using starch and sodium silicate.^17^

We chose BHA as a benchmark for evaluating the collection properties of ASL and LSL because hydroxamic acids generally have better selectivity for REM than fatty acids, though they are adversely affected by fines.^40^ There is a consensus that at low concentrations, hydroxamic acids are adsorbed on bastnäsite through chemisorption.^31,41−43^ At higher concentrations, the adsorption mechanism is surface precipitation (also called “surface reaction”) of hydroxamate complexes with hydroxy cations Ce(OH)^2+^ and Ce(OH)_2_^+^ formed by natural dissolution of this semisoluble mineral.^31,43^ Chemisorption and the surface reaction with surface Fe^III^–OH groups have also been proposed for the adsorption of hydroxamic acid on hematite.^44,45^

This research presents a potential application of the effect of the redox properties of ceria on its flotation behavior. We find that the fully oxidized (Ce^IV^) state adversely affects the flotation of ultrafine ceria against hematite with ASL, LSL, and BHA, while the coexistence of Ce^III^ and Ce^IV^ on the ceria surface allows BHA to selectively float ceria at an acidic pH. ASL and LSL are promising collectors for direct flotation of REM, as well as the reverse flotation of hematite from REM. The selective hydrophobic agglomeration of ceria in LSL flotation destroys the froth. Hence, even though hydrophobic agglomeration is a conventional approach to improve the flotation of fines, it can also be detrimental. We propose a tentative mechanistic interpretation of observed trends based on ζ-potential, contact angle, adsorption density, single-mineral flotation, and X-ray photoelectron spectroscopy (XPS) data.

## 2 Experimental Section

### 2.1 Reagents

Diacetylated LSL and ASL were provided by the Bio Base Europe Pilot Plant, Ghent, Belgium. BHA (98%) was acquired from Alfa Aesar. All of the ligands were used without further purification. NaOH and HNO_3_ were used to adjust pH, while NaNO_3_ was used as a background electrolyte. These three reagents were purchased from VWR. All solutions were prepared in Milli-Q water. Ce(NO_3_)_3_·6H_2_O used for titration experiment was purchased from Merck.

### 2.2 Metal Oxide Particles

Hematite mineral was provided by Rana Gruber, Norway. Quartz particles were the same as in our previous work.^28^ Reduced cerium oxide particles, which we call CeO_2_^(red)^ hereafter, were purchased from VWR. They were used as received. Oxidized cerium oxide particles, which we call CeO_2_^(ox)^ hereafter, were synthesized by sintering commercial 10 nm (99.9%) CeO_2_ nanoparticles provided by Meliorum Technology. The characterization of the nanoparticles by ζ-potential and transmission electron microscopy can be found in Supporting Information (Figure S4). To synthesize CeO_2_^(ox)^, a 60:50 (w/w) mixture of the CeO_2_ nanoparticles with water was placed in a corundum jar and dried overnight at 80 °C, followed by calcination at 1000 °C in air for 3 h with a heating rate of 250 °C/h. The calcination was done in a Nabertherm programmable furnace. The final porous sample was crushed in a hand mill to obtain a visually homogeneous powder, which was used for the further study.

### 2.3 Stock Solutions of Surfactants

A 1 g/L stock solution of ASL was prepared by dissolving the surfactant in water. A 0.6 g/L stock solution of LSL was prepared in 2 mM NaOH due to the low solubility of LSL in water. A 2 g/L solution of BHA was prepared in water. The stock solutions were prepared on the same day that the experiments were conducted.

### 2.4 Specific Surface Area and Particle Size Distribution

Brunauer, Emmett, and Teller (BET) specific surface areas were measured by using a Micromeritics Tristar 3000 Analyzer. The samples were degassed at 300 °C under a helium flow for 4 h. The particle size distribution was measured by the dynamic light scattering method using a Mastersizer 3000E.

### 2.5 XRD

X-ray diffractometry (XRD) was used to determine the mineralogy of the powder samples. For quantitative phase analysis, particles were additionally ground in ethanol using agate milling rods. The measurements were conducted using a Bruker D8 Advance Series 2 XRD instrument with a Co Kα source. The samples were scanned in the 2θ range of 5–80° at a scan rate of 0.02 step/s and a rotation rate of 60 rpm. The XRD peaks were used to determine the crystallinity and preferred orientation of ceria particles.^46^

### 2.6 SEM

The morphology analysis of particles was performed using a Hitachi SU6600 field emission SEM. A copper double-sided tape was used as a support. Metal oxide particles were placed on individual tapes, followed by a carbon coating to eliminate charging.

### 2.7 X-ray Photoelectron Spectroscopy

XPS measurements were conducted using a Kratos Axis Ultra DLD spectrometer (Kratos Analytical, UK) with a monochromatized Al X-ray source (*h*υ = 1486.69 eV) operating at a power of 100 W and a low energy electron beam charge-neutralization flood gun. The regional spectra were measured at a pass energy of 80 eV and a resolution of 1 eV. Each regional and survey spectrum was accumulated for 6 min. The binding energy scale was calibrated by setting the C 1s peak of adventitious sp^3^ carbon at 285.0 eV.

XPS spectra were measured on the CeO_2_ particles washed in water at pH 4 (adjusted by HNO_3_) for 2 h, separated by centrifugation, and dried in a desiccator. The dry particles were pressed into the freshly exposed surface of an indium foil (>99% purity, Sigma-Aldrich), and the foil was attached to the XPS holder.

### 2.8 Raman Spectroscopy

Raman spectra were measured by using a WITec Alpha 300R imaging system with a laser wavelength of 532 nm and a power range of 0.1–66 mW.

### 2.9 Zeta Potential

Zeta (ζ) potential was measured using a Malvern ZetaSizer Nano Z (laser Doppler microelectrophoresis) instrument. The metal oxide particles were first dispersed at 0.2 wt % in 0.001 M NaNO_3_ by sonicating for 10 min. The pH-adjusted samples were equilibrated on a shaking table overnight. Afterward, the pH was readjusted with 0.01 M NaOH and HNO_3_ solutions and measured for each sample before measuring its ζ-potential. As the material settings in ZetaSizer, we used refractive indices of 3.0 and 2.2 and absorption of 0.8 and 0.5 for iron and cerium oxides, respectively. Each ζ-potential data point is an average of 3 replicate points where each point was an average of 10 scans each. The ζ-potential uncertainty is ±2 mV, and the pH uncertainty is within 0.1 units.

### 2.10 Precipitation of Basic Ce^III^-Collector/OH Complexes

A solution containing 100 μM Ce(NO_3_)_3_·6H_2_O and 100 μM collector was titrated under open air conditions with 0.1 M NaOH from pH 4.2 to 10. Precipitation was visually observed at pH 8.5–9. In the BHA and ASL solutions, precipitation was accompanied by a pH decrease by 6–7. In the case of LSL, pH decreased only by 0.1–0.3. The pH decrease upon complexation of the collector headgroups with Ce^III^ suggests that they are coordinated, releasing protons. As a control, we titrated 100 μM Ce(NO_3_)_3_·6H_2_O. The formation of the precipitate was accompanied by a pH increase. Base precipitation of Ce^III^ typically produces Ce^IV^ hydroxide due to the fast oxidation of Ce^III^ by dissolved oxygen.^47^ The ASL and LSL precipitates were colorless, while the BHA precipitates were dark yellow.

### 2.11 Adsorption Density Using Total Organic Carbon

A HACH DR3900 spectrophotometer was used for TOC analysis to determine the adsorption density of ASL and LSL on hematite. The differential method was used to calculate the TOC, by measuring total carbon (TC) and subtraction of measured total inorganic carbon (TIC). Metal oxide particles (0.5 g) were dispersed in a 50 mL volume of 100 μM surfactant solutions at pH 4. The pH was readjusted after 30 min and then equilibrated on a shaking table for 2 h. The final dispersions were filtered through a 0.45 μm syringe filter, and the filtered solutions were analyzed. TOC was measured using a test kit HACH 2–65 mg/L. Test tubes with the filtered solutions were heated up to 120 °C in a thermostat for 2 h. The coloration of indicators was measured by the spectrophotometer. Each point of adsorption density is an average from two replicates. The adsorption density was normalized by the BET surface area and converted to the number of adsorbed monolayers using the minimum surface areas (*A*_min_) of ASL and LSL at the air–water interface of 88 Å^2^/ molecule and 94 Å^2^/ molecules, respectively (Section S1).

### 2.12 Wettability

The Washburn method was used to assess the hydrophobicity of CeO_2_^(red)^, CeO_2_^(ox)^, and α-Fe_2_O_3_ after conditioning in water and 100 μM solutions of ASL, LSL, or BHA, all at pH 4. This method is based on the capillary rise of a liquid through a packed bed of particles in a capillary tube. The measurements were conducted using a Biolin Scientific Sigma 702 instrument at a 0.5 g loading of particles into the tube. Each measurement was at least triplicated, and the average contact angle is reported. In the adsorption experiment, 3 g of metal oxide was added to a 200 mL solution, followed by stirring for 2 h. The final suspension was filtered and dried at room temperature. Due to the strong hydrophobicity of CeO_2_^(red)^ after adsorption of ASL and LSL at pH 4, its wettability was observed visually by depositing a water drop on the particle bed using a high-speed camera Chronos 1.4, Kron Technologies Inc. (Section S6).

### 2.13 Flotation

Flotation was conducted using a XFG II flotation machine at the surfactant concentration of 100 μM. We did not use a frother in the case of biosurfactants due to their good intrinsic foaming properties.^28^ As BHA lacks these properties, we used 50 μM DowFrothTM 200 as a frother in the flotation with BHA.

**Figure 2:**
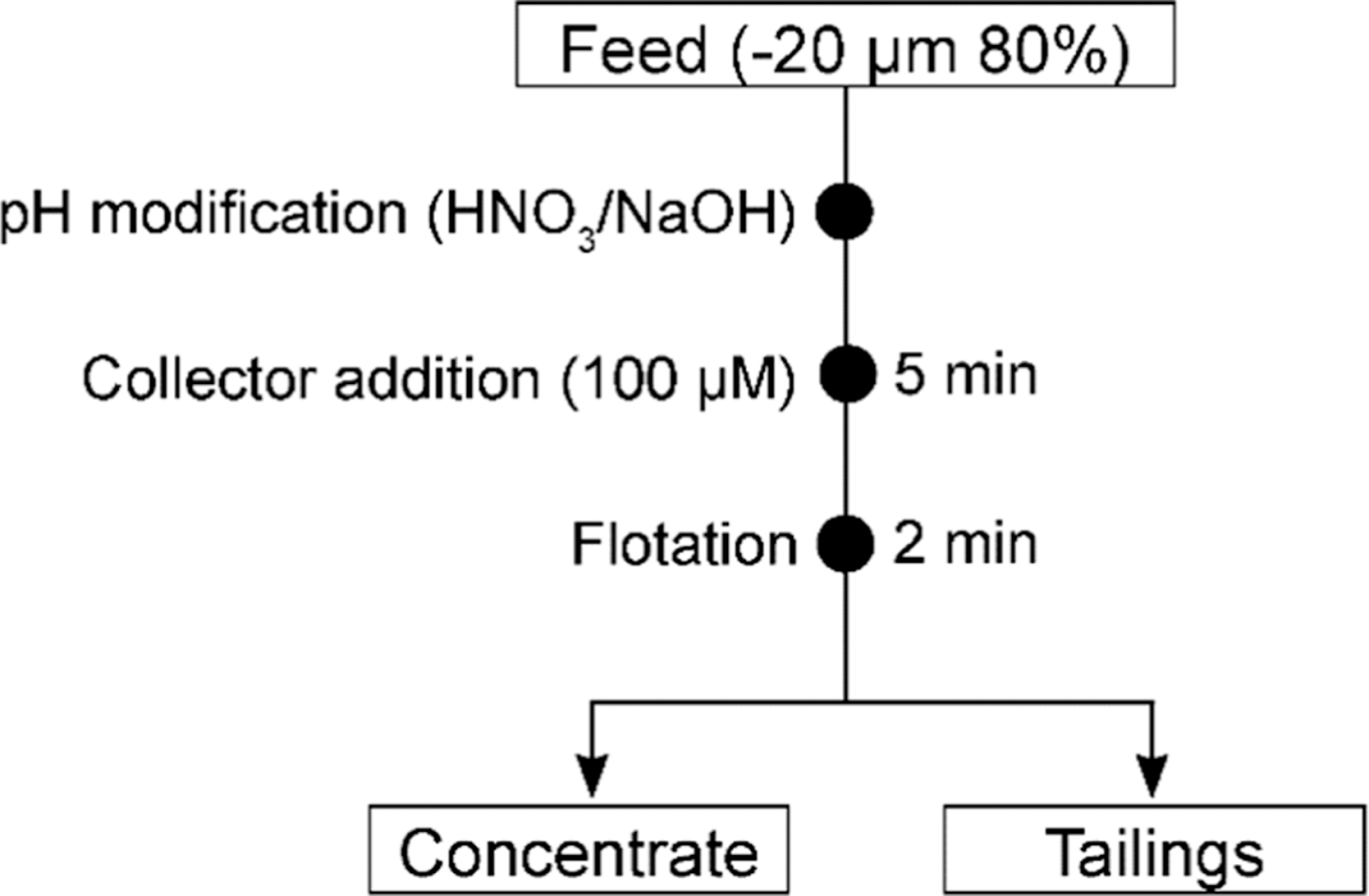
Flotation flowsheet.

Flotation conditions are listed in Figure 2. For single mineral flotation, 1 g of CeO_2_ or α-Fe_2_O_3_ particles was added to a 100 mL flotation cell. Then, 90 mL of Milli-Q water was added, and the pulp was stirred for 2 min. After 2 min, the pH was adjusted to the required values and the surfactant was added from the stock solution with the pH between 5 and 6, and pH was readjusted after 4 min, followed by the flotation in 1 min. All of these steps were performed with continuous stirring of 1500 rpm. The flotation time was 2 min at an airflow rate of 100 L/h (∼1650 mL/min). The floated and nonfloated fractions of metal oxide particles were collected and filtered using a filter paper of the 589/3 grade, followed by drying in a heating chamber. All of the flotation tests were repeated twice, and the average values were reported. The same procedure was repeated in two- and three-mineral flotation. The ratios of the metal oxides were 1:1 and 1:1:1, and the weight of each oxide was 0.5 g.

The weight of the dried particles was used to determine the flotation recovery. Recovery was calculated as the weight percentage of recovered to recovered plus rejected particles. The selectivity of flotation was characterized using a grade. To calculate the grade, the target and gangue metal percentages were converted to their respective oxides. Grade was then determined by calculating the percentage of target metal oxide in relation to the total metal oxide percentage, which includes both target and gangue metals in the recovered portion. The grade was estimated by using a portable X-ray fluorescence (XRF) instrument. Each experiment was duplicated, and the average values are reported. The deviation of the duplicate results from the average was below 10%.

## 3 Results and Discussion

This section is organized as follows. First, the biosurfactants and ultrafine hematite and ceria particles studied were characterized. The biosurfactants were characterized by static surface tension (S1 of Supporting Information), while the metal oxide particles are characterized using XRD, XPS, Raman, and BET and particle size distribution (S3 of Supporting Information). Next, the effect of the ceria redox state on the performance of the biosurfactants and BHA was studied using mini-flotation tests on single and multimineral systems. Based on zeta potential, adsorption density, and hydrophobicity data, the results were explained in terms of the interactions of the metal oxides with the three collectors.

### 3.1 Characterization of Ultrafine Metal Oxide Particles

Based on XPS and Raman analysis (S3 of Supporting Information), CeO_2_^red^ is partially reduced, while CeO_2_^ox^ is fully oxidized. Furthermore, XRD confirms that CeO_2_^red^ and CeO_2_^ox^ have the same polycrystalline cubic ceria structure and do not contain other phases (S3 of Supporting Information). It allows one to compare the effect of the redox state of the ceria particles, meaning the effect of the reduced (Ce^3+^/C^4+^) and oxidized surface on the interaction with the biosurfactants and BHA. XRD of the hematite particles confirms their 99% purity, while XPS detects only traces of Si (Figure S12a). The complete characterization of the particles can be found in Supporting Information. The particle size distribution shows that all particles are ultrafine with *D*_80_ < 20 μm. Particles with this size distribution were used in all analyses and flotation tests.

### 3.2 Effect of Ceria Redox State on Collector Performance

#### 3.2.1 Single-Mineral Flotation

**Figure 3:**
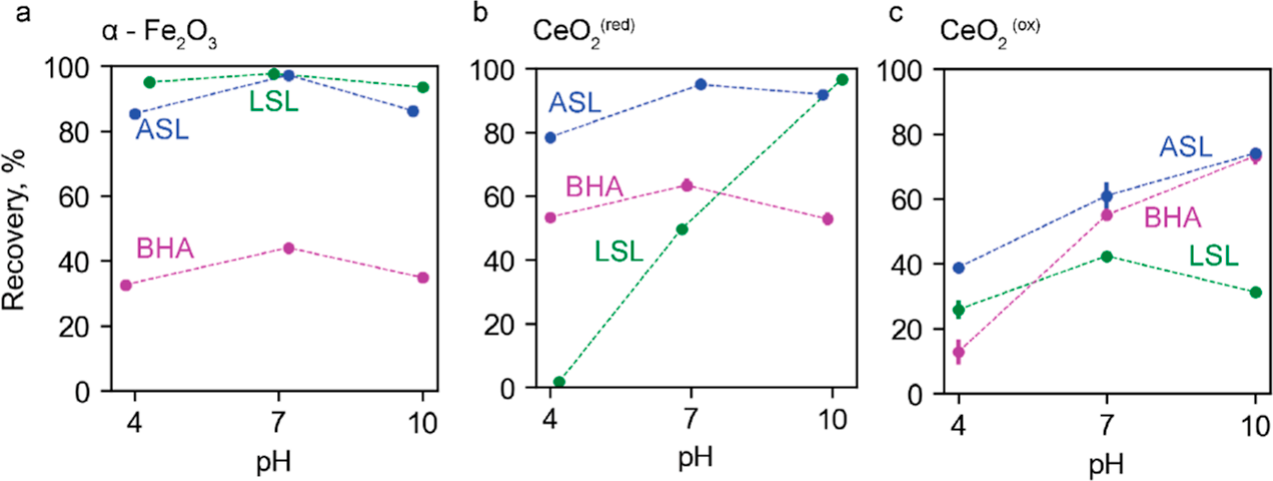
Effect of pH on the single-mineral flotation of (a) α-Fe_2_O_3_ (b) CeO_2_^(red)^, and (c) CeO_2_^(ox)^ with 100 μM LSL, ASL, and BHA. 50 μM DawFroth200TM was used as a frother with BHA. The reported recovery is average from duplicate with an error no more than 10% recovery value.

To compare the collector-induced hydrophobicities of the metal oxides under flotation conditions, we performed single flotation tests. We keep in mind that for ultrafine particles, this method is prone to the interferences of hydrophilic entrainment/entrapment.^29^ The tests were run at collector concentrations of 100 μM and a conditioning time of 5 min (Section 2.13).α-Fe_2_O_3_: As shown in Figure 3a, both ASL and LSL at 100 μM float 90–100% of hematite over a wide pH range from 4 to 10, while 100 μM BHA floats only 35–45%. In all cases, the pH dependence is not pronounced, with a weak maximum at pH 7. For comparison, at 50 μM, ASL flotation increases linearly from 30 to 70%, as pH increases from 4 to 10, while LSL flotation has a pronounced maximum of 70% at pH 4–6.^28^ Since ASL adsorbs on hematite in a hydrophilic (tail dangling) configuration at 50 μM and acidic pH,^28^ the significant improvement in the hydrophobicity of the ASL adlayer at 100 μM indicates that the molecule switches to the hydrophobic ring configuration (with both its head groups coordinated to the hematite surface) with increasing surface coverage. The improvement in the hydrophobicity of the LSL adlayer with increasing surface coverage suggests that the packing of the molecules is improved.

The poor flotation of hematite with BHA at pH 7 and 10 (Figure 3a) contradicts the maximum flotation with 200 μM octylhydroxamic acid at pH 9.^48^ This contradiction can be tentatively explained by the short (2 min) preconditioning time before the 5 min conditioning with the collector in our tests (Section 2.13). Increasing both the preconditioning and conditioning times significantly improves the floatability of hematite with octylhydroxamic acid.^48^ This phenomenon can be rationalized by the fact that hydroxamic acids require hydroxylated metal cations to be adsorbed on a mineral surface.^31,43−45^ As shown by XPS, the preconditioning in water increases the level of hydroxylation of the hematite surface (Figure S12c).

*CeO*_*2*_^*(red)*^: The floatability of CeO_2_^(red)^ with BHA at pH 4–10 is 55–65% (Figure 3b), which is 20% higher than that of hematite (Figure 3a). As in the case of hematite, there is a weak maximum at pH 7. In comparison, hydroxamic acids are most effective in the flotation of bastnäsite at pH 8–9.^3,17,43,49^ The extension of the floatability of CeO_2_^(red)^ with BHA into the acidic pH range can be attributed to the presence of Ce^IV^ on the ceria surface. According to the commonly accepted model,^42^ hydroxamic acids are dissociatively coordinated by the positively charged metal–OH species on the mineral surface or in the solution. Ce^III^ and Ce^IV^ form such hydrolyzed cations at pH above 6 and below 4.5, respectively.^50,51^ The lower pH of hydroxylation of Ce^IV^ ions is explained by their higher acidity and ionic charge. This effect is important because it allows for decoupling of the hematite and ceria flotation maxima (Section 3.2.2).

Compared to BHA, ASL imparts a much higher hydrophobicity to CeO_2_^(red)^ and floats 80–90% of CeO_2_^(red)^ with a weak maximum at pH 7 (Figure 3b).

In contrast to BHA and ASL flotation, LSL flotation is highly pH-dependent. The recovery is less than 5% of CeO_2_^(red)^ at pH 4, increasing to 50% and 95% at pH 7 and 10, respectively (Figure 3b). The highest floatability of 95–98% is achieved by ASL and LSL at pH 7–10 and 10, respectively.

The low recovery of CeO_2_^(red)^ by LSL at pH 4 is explained by the froth instability in this system (Figures S6 and 7b). In Section S5, we argue that the defoaming effect in the LSL flotation of CeO_2_^(red)^ is caused by the formation of large (50–100 μm) compact hydrophobic CeO_2_^(red)^ aggregates. The defoaming properties of hydrophobic particles in this size range have been demonstrated in the flotation of quartz particles.^10^ Both the “dynamic” and the “static” froth stability are maximized by a 26–44 μm particle size fraction that has an intermediate degree of hydrophobicity (corresponding to a contact angle of 65° for a water sessile drop on a quartz plate). More hydrophilic particles did not affect the froth properties, but more hydrophobic particles destabilized the froth. For larger particles (74–106 μm), these effects are much less pronounced.^10^ Froth stability in coal flotation depends on the hydrophobicity and the proportion of fine coal particles (Ref (7) and references therein).

CeO_2_^(ox)^: Figure 3c shows that the oxidized state significantly degrades the floatability of ceria with all three collectors in the whole pH range. The exception is BHA flotation at pH 10, which recovers 75% of CeO_2_^(ox)^. The recovery of CeO_2_^(ox)^ with ASL increases with pH from 40% to 75%. The recovery of CeO_2_^(ox)^ with LSL is 25–40% with a weak pH dependence. In contrast to CeO_2_^(red)^, CeO_2_^(ox)^ produces some froth in LSL flotation at pH 4.

As CeO_2_^(red)^ and CeO_2_^(ox)^ have not only a different redox state (Section 3.1) but also a different morphology (Section 3.1.), we tested the effect of oxidation on the floatability of CeO_2_^(red)^ with the biosurfactants. These tests confirm that the main control of the interaction of ceria with the biosurfactants is its oxidation state (Section S7).

#### 3.2.2 CeO_2_^(red)^- α-Fe_2_O_3_ Binary System

**Figure 4:**
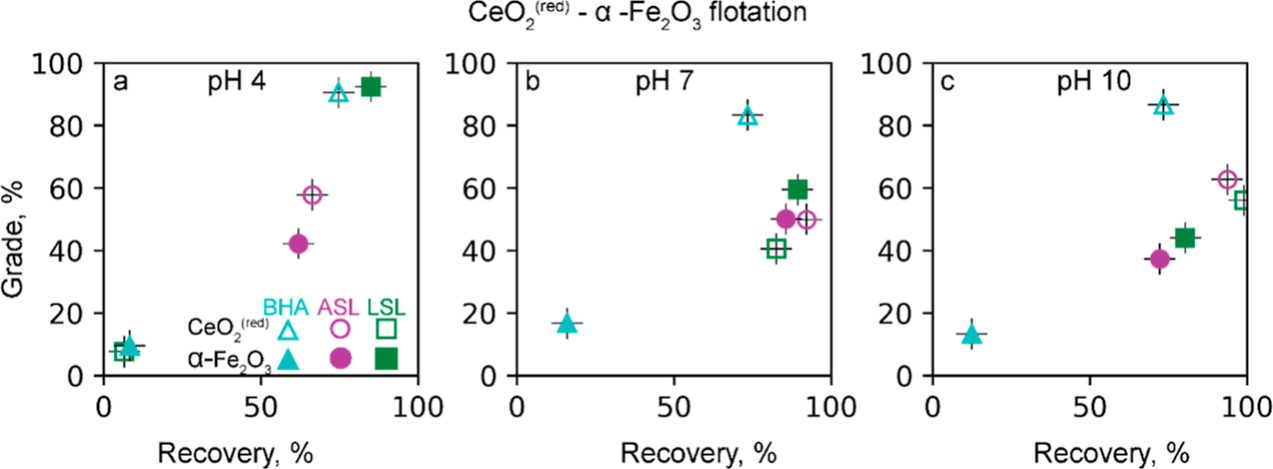
Recovery and grade of CeO_2_^(red)^ and hematite ultrafine particles floated by ASL, LSL, and BHA at (a) pH 4, (b) pH 7, and (c) 10. A froth agent DowFroth200TM 50 μM concentration was used together with BHA. The reported values are average from duplicate with an error no more than 10% of recovery and grade values.

The highest selectivity to CeO_2_^(red)^ over hematite at pH 4, 7, and 10 is achieved by BHA (Figure 4). The average grade of CeO_2_^(red)^ in the BHA flotation is 85–95% with a recovery of 75% at pH 4, 7, and 10. The highest grade (and recovery) is observed at pH 4, where BHA recovers 74% ceria at a 90% grade.

The highest recovery and grade of CeO_2_^(red)^ at pH 4 is surprising considering that (i) hydroxamic acids form much more stable complexes with Fe^III^ than with Ce^III^^3^ and (ii) the bastnäsite flotation with hydroxamic acids is maximal at pH 8–9.^3,17,43,49^ This result can be explained by a favorable combination of two effects observed in single mineral flotation: At pH 4, BHA barely floats hematite but floats 50–60% CeO_2_^(red)^ (Figure 4a,b). The former effect can be explained by the slow kinetics of the hydroxamic acid adsorption on hematite.^44^ The floatability of ceria at pH 4 is explained by the presence of Ce^IV^ on the ceria surface, which promotes the formation of the Ce–OH adsorption sites at this pH. However, the copresence of Ce^III^ is also important (Section 3.1.).

Conversely, the highest selectivity to hematite vs CeO_2_^(red)^ is achieved by LSL at pH 4 (Figure 4a), where the grade and recovery is 85–95%. Given that the froth stability in this system is intermediate (Figure S6c), the selective rejection of CeO_2_^(red)^ particles can be explained by their selective hydrophobic agglomeration to the large nonfloatable size (Section 3.3.1). The selectivity of LSL to hematite is suppressed with increasing pH (Figure 4b,c), which is due to the increase in the floatability of CeO_2_^(red)^ with LSL (Figure 3b).

ASL is nonselective and floats both CeO_2_^(red)^ and hematite under the conditions tested, consistent with the similarly high floatability of both metal oxides in the single-mineral flotation (Figure 3b).

#### 3.2.3 CeO_2_^(ox)^- α-Fe_2_*O*_3_ Binary System

In line with the single-mineral flotation results (Section 3.3.1.), the oxidized state of ceria adversely affects its separation from hematite for all three collectors.

**Figure 5:**
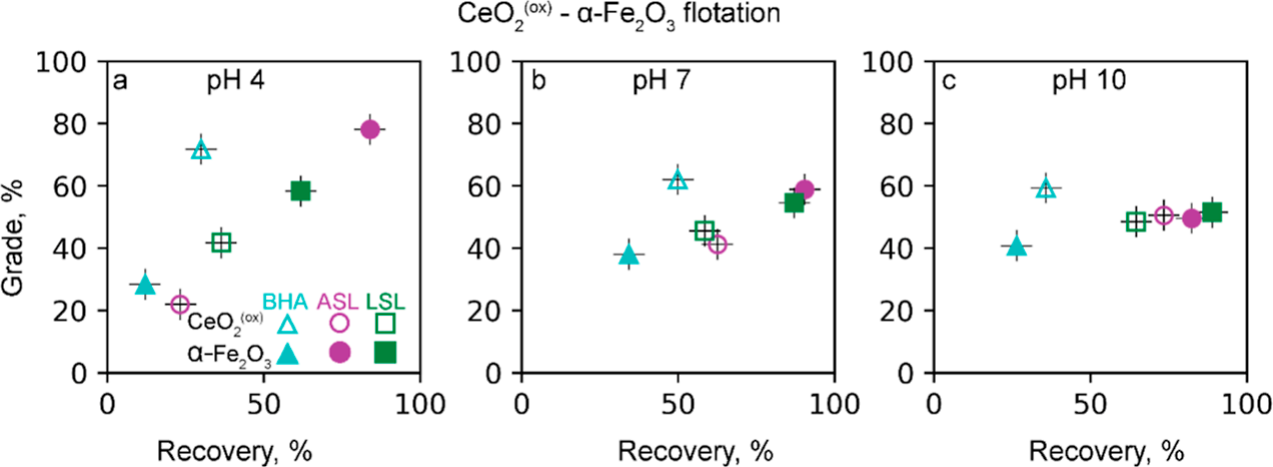
Recovery and grade of CeO_2_^(ox)^ and hematite ultrafine particles floated with ASL, LSL, and BHA at (a) pH 4, (b) pH 7, and (c) 10. A froth agent DowFroth200TM 50 μM concentration was used together with BHA. The reported values are average from duplicate with an error no more than 10% of recovery and grade values.

Even though BHA remains the best collector of ceria, the grades and even more so the recoveries decrease compared to those of CeO_2_^(red)^ (Figure 5). The maximum CeO_2_^(ox)^ grade of 75% is observed at pH 4 (vs 90% for CeO_2_^(red)^). At pH 7 and 10, the grade decreases to 60% (vs 85–90% for CeO_2_^(red)^). The CeO_2_^(ox)^ recoveries at pH 4, 7, and 10 are only 30, 50, and 40%, respectively (vs 70% for CeO_2_^(red)^). The more pronounced suppression of the ceria recovery versus grade suggests that the detrimental effect of the oxidized state originates from the low surface coverage of BHA, which can be related to the low concentration and low basicity of the surface hydroxyls (Section 3.1.).

At pH 7 and 10, both ASL and LSL are selective. At pH 4, LSL changes from a highly (95%) selective collector of hematite to a weakly (60%) selective collector of ceria (Figure 5a). Instead, the best collector of hematite at pH 4 becomes ASL (grade and recovery are 80%). This result is consistent with the 85% floatability of hematite with ASL versus the 40% floatability of CeO_2_^(ox)^ in single-mineral flotation (Figure 3a,c).

#### 3.2.4 ASL and LSL Flotation of Three-Mineral System CeO_2_^(red)^–αFe_2_O_3_–SiO_2_

ASL and LSL were further tested in the flotation of CeO_2_^(red)^, α-Fe_2_O_3_, and SiO_2_ in a 1:1:1 ratio (0.5 g each). The remaining parameters were the same as for the binary flotation systems. Before reporting the results, it should be noted that both biosurfactants do not float quartz in the single-mineral flotation.^28^

**Figure 6:**
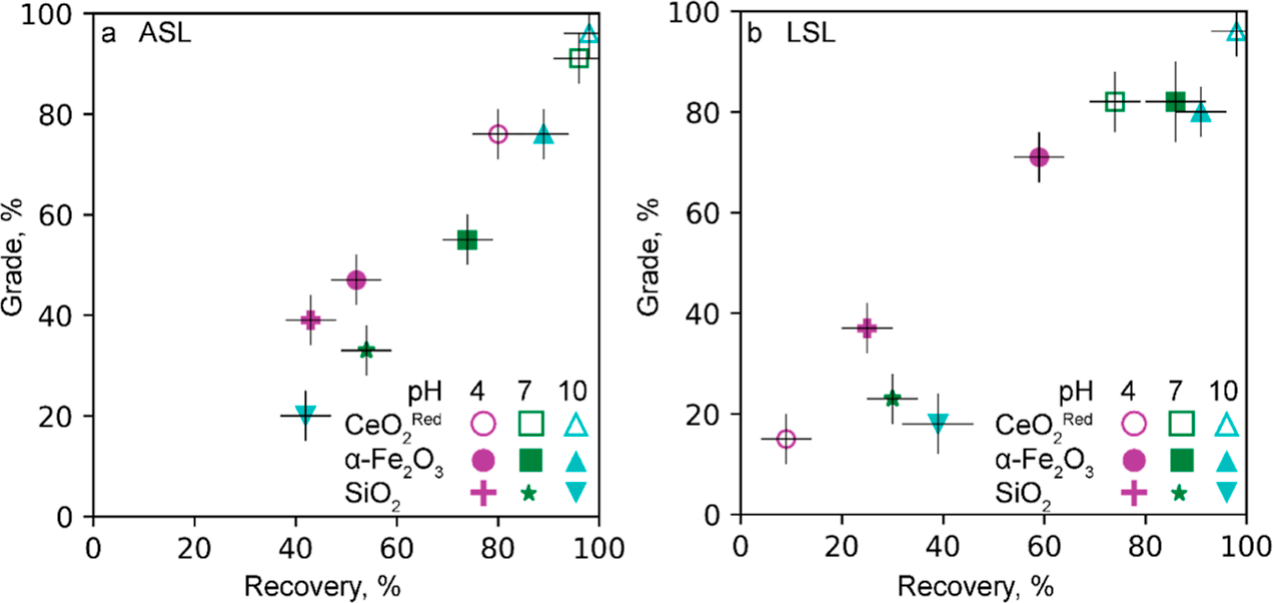
Flotation of three-mineral system CeO_2_^(red)^–α-Fe_2_O_3_–SiO_2_ with 100 μM of (a) ASL and (b) LSL at pH 4, 7, and 10. The reported values are average from duplicate with an error no more than 10% of recovery and grade values.

At pH 10, both ASL and LSL separate α-Fe_2_O_3_ and CeO_2_^(red)^ as a group from SiO_2_ (Figure 6). They recover 96% CeO_2_^red^ and 76–80% hematite versus 40% quartz. This result is consistent with the CeO_2_^red^-hematite and hematite-quartz flotation at pH 10 shown in Figure 4c and Ref (28), respectively. The best selectivity to ceria is achieved by ASL at pH 7, where the grade of ceria is 92% versus 55% and 33% of hematite and quartz, respectively (Figure 6a). As in the case of the binary α-Fe_2_O_3_–CeO_2_^red^ system (Figure 4), LSL is selective toward α-Fe_2_O_3_ (72% grade) at pH 4, while the grade of SiO_2_ is ca. 38% and that of CeO_2_^(red)^ is only ca. 15% (Figure 6b). These results demonstrate that ASL and LSL at pH 4 and 10 have the potential to separate REM from iron oxides and silicates.

### 3.3 Interaction of Collectors with Ultrafine Metal Oxides

#### 3.3.1 Adsorption Density of ASL and LSL at pH 4

To interpret the significant contrast in the floatability of the metal oxides at a biosurfactant concentration of 100 μM and pH 4 (Section 3.2.1. and Section 3.2.2.), we measured their adsorption density under these conditions. The adsorption densities were converted to a monolayer coverage using the minimum surface areas (*A*_min_) of ASL and LSL at the air–water interface of 88 Å^2^/molecule and 94 Å^2^/molecule, respectively (Sections 2.11 and S1).

As shown in Table 1, hematite adsorbs 0.8 and 1.2 formal monolayers of ASL and LSL, respectively. At the same time, both cerias adsorb less than half of a monolayer. The adsorption density on CeO_2_^(red)^ is by 30–80% higher than that on CeO_2_^(ox)^. Thus, the affinity of the metal oxides for the biosurfactants at pH 4 is on the order of α-Fe_2_O_3_ > CeO_2_^(red)^ > CeO_2_^(ox)^.

**Table 1:** Adsorption Density of ASL and LSL on α-Fe_2_O_3,_ CeO_2_^(ox)^, and CeO_2_^(red)^ Interacted with 100 μM Solutions of the Surfactants at pH 4

	α-Fe2O3		CeO_2_^ox^		CeO_2_^red^	
	ASL	LSL	ASL	LSL	ASL	LSL
adsorption density, μM/m^2^	30.0 ± 0.5	44.0 ± 0.5	8.1 ± 0.6	12 ± 3	14 ± 3	16 ± 2
formal monolayer	0.8	1.2	0.21	0.34	0.37	0.45

The higher affinity of CeO_2_^(red)^ versus CeO_2_^(ox)^ to the biosurfactants is in line with the general notion that O-ligands more easily coordinate to a partially reduced versus stoichiometric CeO_2_ surface, which is associated with FLPs on the former.^52^ In catalysis, these adsorption sites are called catalytic hotspots.^53^ In the gas phase, carboxylic acids and other O-donors are adsorbed dissociatively and more strongly on reduced ceria than on the stoichiometric one.^36,54^ XPS shows that CeO_2_^(red)^ is more hydroxylated compared to CeO_2_^(ox)^ and its surface hydroxyls are more basic (Section 3.1), suggesting that the collectors are more strongly adsorbed on the sites containing basic hydroxyls. This conclusion is supported by the much higher floatability of CeO_2_^(red)^ compared to that of CeO_2_^(ox)^ (Section 3.2.1).

#### 3.3.2 Zeta (ζ) Potential of Ceria and Hematite

The ζ-potential analysis (the electrostatic potential at the shear planes) is a conventional indirect technique used to study the adsorption of collectors/surfactants on mineral particles.^45,55^ It is related to the surface charge of the particles, which typically responds differently to the chemisorption and physisorption of anionic collectors. We used this technique to characterize the adsorption of ASL, LSL, and BHA on ultrafine ceria and hematite particles. This study is performed in 1 mM NaNO_3_ at the collector concentration of 100 μM. This concentration is slightly above and below the CMC at pH 4 of LSL and ASL, respectively.

**Figure 7:**
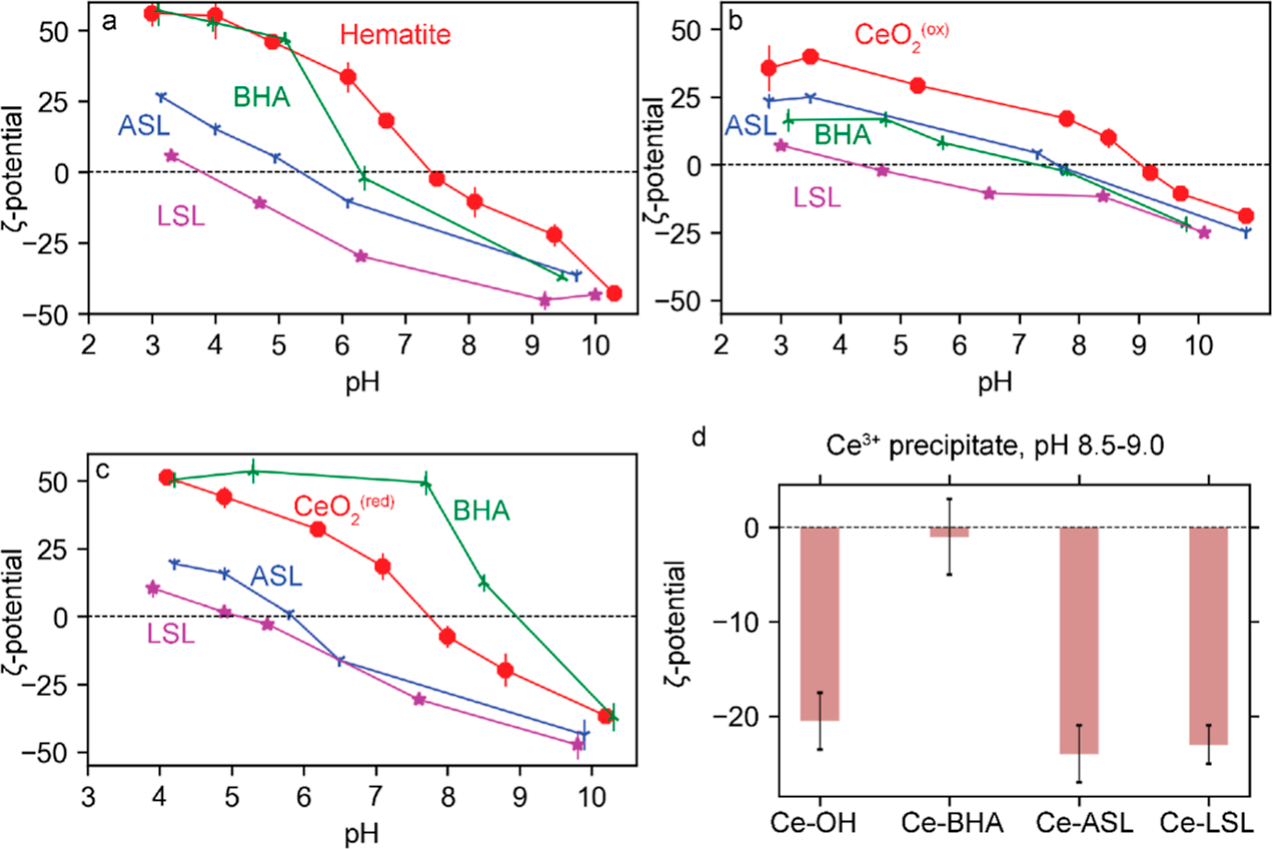
ζ-potential of (a) hematite, (b) CeO_2_^(ox)^, and (c) CeO_2_^(red)^ in the absence and presence of 100 μM LSL, ASL, and BHA as a function of pH; (d) basic Ce precipitates in the absence and presence of LSL, ASL, and BHA measured at pH 8.5–9.0. The preparation of these precipitates is described in Section 2.10. The background solution is 1 mM NaNO_3_. The conditioning time is 12 h. The vertical error bars (from triplicates) are 5 mV or less.

*Hematite*: The iso-electric point (IEP) of 7.5 of hematite in the absence of a collector is consistent with reported values (Figure 7a).^56^

All three collectors make the ζ-potential of hematite negative at pH 7.5, shifting IEP to lower values. The strongest impact is caused by LSL (IEP = 3.8), followed by ASL (IEP = 5.5) and BHA (IEP is 6.5).

ASL shifts the ζ-potential of hematite at pH 4 and 9 from +55 to +25 mV and from −20 to −30 mV, respectively. The significant negative charging of hematite at pH above IEP suggests that ASL is chemisorbed in the anionic form.^28^ Compared to anionic ASL, nonionic LSL makes hematite even more negatively charged (Figure 7a). The ζ-potential of hematite in the LSL solutions at pH 4 and 9 is ca. 0 and −33 mV, respectively. This effect can be explained by the adhesion of LSL colloids and chemisorption of LSL in the anionic form at acidic and basic pH, respectively.^28^

The effect of BHA on the ζ-potential of hematite is qualitatively similar to that of octylhydroxamic acid (Figure 7a).^44,45^ The exception is the pH range of 3–5, where BHA does not affect ζ-potential (Figure 7a). The generally accepted mechanism of the hydroxamic acid adsorption on hematite is chemisorption combined with surface precipitation at higher ligand concentrations.^44,45^ Both the reactions include the formation of 5-membered chelating complexes between a hydroxamate anion and Fe^III^ through the interaction of hydroxamic acid with the Fe^III^–OH species.^44,45^ The inconsistency at pH 3–5 can be explained by the dominance of surface precipitation of neutral Fe(BHA)_3_ complexes promoted by the long conditioning time (12 h). This explanation is supported by (i) the onset of hematite solubility around pH 4, (ii) the significant increase in the hydroxylation of the hematite surface after conditioning at pH 4 for 12 h (Figure S12c), and (iii) the formation of insoluble Fe(BHA)_3_ complexes at pH > 3.^44^ Furthermore, the extrapolation of the ζ-potential of the Fe(BHA)_3_ complexes^44^ suggests that they have an IEP around 4.

*Ceria*: The IEP of CeO_2_^(ox)^ and CeO_2_^(red)^ is 9.0 and 7.5, respectively (Figure 7b,c), within the pH range of 6–10 reported for ceria.^57−59^ This difference is consistent with a report that nonhydrated ceria is positively charged and develops a more negative surface charge (accompanied by a decrease in IEP) with the hydration time due to the replacement of adsorbed protons H^+^ by OH^–^ ions.^60^ We exclude organic impurities as a possible reason for the difference since there is no effect of acid washing on the ζ-potential of the ceria (Figure S5).

BHA shifts the IEP of CeO_2_^(ox)^ from 9.0 to 7.7 (Figure 7b). In contrast, it increases the IEP of CeO_2_^(red)^ from 7.5 to 9.0 (Figure 7c). An increase in IEP has also been observed upon the adsorption of BHA on bastnäsite.^49^ Considering that the ζ-potential of basic Ce-BHA precipitates is almost zero at pH 9.0 (Figure 7d), the increase in IEP can be explained by the dominance of surface precipitation in the adsorption mechanism of BHA on CeO_2_^(red)^ at a basic pH.

LSL decreases the IEP values of CeO_2_^(ox)^ and CeO_2_^(red)^ to 4.2 and 5.0, while ASL decreases to 7.5 and 5.8, respectively (Figure 7b,c). As in the case of hematite, the shift of the IEP of ceria to more acidic values indicates that ASL and LSL are chemisorbed in the anionic form. This pathway at basic pH can be accompanied by surface precipitation of Ce-biosurfactant complexes given that basic Ce^III^–OH, Ce^III^-ASL, and Ce^III^-LSL precipitates are negatively charged at pH 9 (Figure 7d). In the case of LSL, there can be an additional contribution of adhered LSL colloids.^28^

Over a wide pH range, ASL, LSL, and BHA are chemisorbed on the metal oxides in the anionic form, which can be accompanied by surface precipitation. In particular, surface precipitation is the dominant adsorption mechanism in the BHA-hematite system at pH 3–5. This mechanism is favored by the hydration of hematite.

#### 3.3.3 Washburn Contact Angle and Spreading of Water Droplets at pH 4

**Figure 8:**
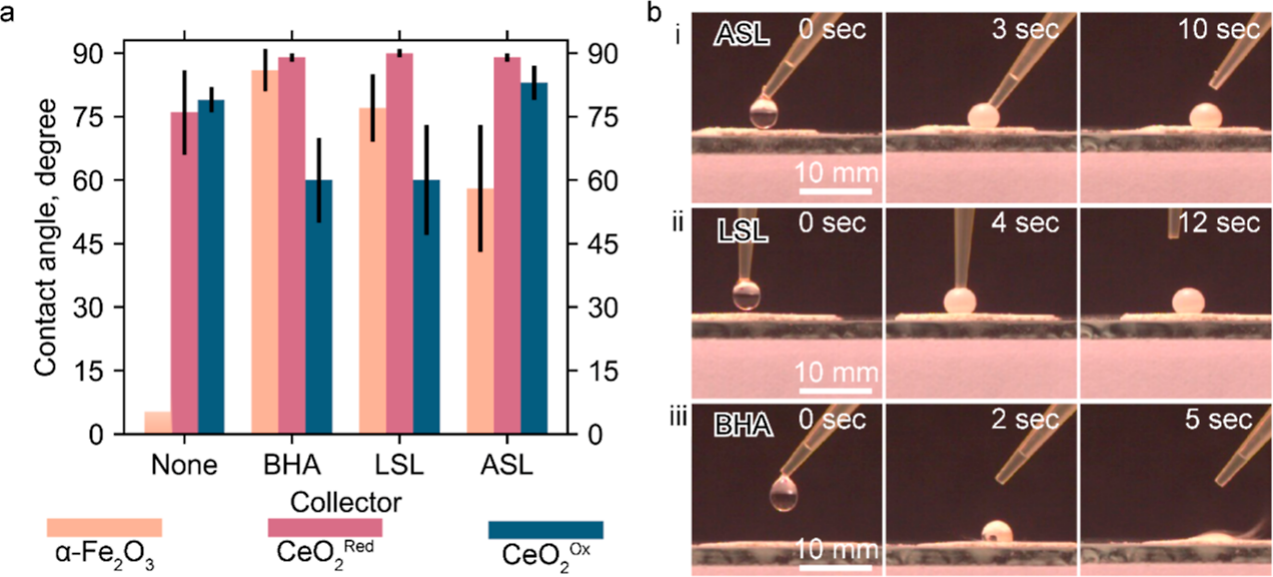
Effect of BHA, ASL, and LSL on (a) Washburn contact angle of CeO_2_^(red)^, CeO_2_^(ox)^, and α-Fe_2_O_3_ particles after 2 h conditioning in 100 μM ligand solutions at pH 4; (b) high-speed camera images of a water drop placed on the bed of CeO_2_^(red)^ particles treated 2 h in 100 μM of (i) ASL, (ii) LSL, and (iii) BHA at pH 4.

Hematite conditioned in water at pH 4 for 2 h is hydrophilic, as evidenced by its Washburn contact angle below 5° (Figure 8a). All three collectors at a concentration of 100 μM render hematite hydrophobic. The strongest hydrophobicity is rendered by BHA, followed by LSL and ASL with contact angles of 80, 75, and 58°, respectively. Particles with contact angles below 60° are typically considered hydrophilic in flotation, as they may require a collector to facilitate flotation. Given that the surface coverage of hematite by ASL is 0.8 monolayer (Table 1), its hydrophilicity suggests that, at least in part, ASL is adsorbed on hematite under the above conditions by one headgroup while the other one is dangling in solution.^28^ The moderate hydrophobicity of the 1.2 monolayer of LSL on hematite (Table 1) suggests that LSL in the second monolayer is adsorbed by hydrophobic interactions, exposing its sophorose group to the solution. This result is also consistent with the adhesion of negatively charged LSL colloids to the positively charged hematite surface.^28^ The relatively high hydrophobicity of BHA-adsorbed hematite indicates that the precipitated Fe(BHA)_3_ complexes (Section 3.2.1) are hydrophobic.

In contrast to hematite, both CeO_2_^(red)^ and CeO_2_^(ox)^ conditioned in water at pH 4 are partially hydrophobic, as manifested by their Washburn contact angles of 77 ± 3° (Figure 8a). This result agrees with the intrinsic hydrophobicity of rare earth oxides.^59,61^ The adsorption of 0.2 monolayers of ASL (Table 1) does not affect the contact angle of CeO_2_^(ox)^. Surprisingly, BHA and LSL make CeO_2_^(ox)^ more hydrophilic, as seen from a decrease in the Washburn contact angle from 75 to 80° to 60 ± 10°. This result can suggest that BHA and LSL are adsorbed on CeO_2_^(ox)^ through hydrophobic interactions, as generally proposed for the surfactant adsorption on hydrophobic surfaces.^55^ In this system, adsorption energy is gained by displacing interfacial water molecules. Alternatively, CeO_2_^(ox)^ can oxidize collectors, given that CeO_2_ can oxidize alcohols, aldehydes, ketones, ethers, and carboxylic acids in the gas phase and the reducing properties of sophorose.^36,62^ We are conducting a detailed spectroscopic study to resolve this dilemma.

In striking contrast, all three collectors increase the Washburn contact angle of CeO_2_^(red)^ to 88–90°. However, these values are inaccurate, as the Washburn method is limited by 90°. Therefore, we studied the spreading of water droplets over CeO_2_^(red)^ particle beds by depositing a water droplet and monitoring its behavior with a high-speed camera (Section S6). In the case of BHA, a water droplet spreads over and permeates the particle bed (Figure 8b(iii)), indicating that the BHA-adsorbed hematite is moderately hydrophobic. In contrast, droplets bounce from the ASL- and LSL-treated CeO_2_^(red)^ (Figure 8b(i,ii); video in Section S6), which indicates that the particle surfaces are highly hydrophobic.

Thus, at pH 4, ASL, LSL, and BHA increase hydrophobicity of CeO_2_^(red)^ but decrease hydrophobicity of CeO_2_^(ox)^, except for the CeO_2_^(ox)^-ASL system where ASL does not have any significant impact. The ASL- and LSL-adsorbed CeO_2_^(red)^ are much more hydrophobic than the BHA-adsorbed counterpart.

## 4 Discussion

Trivalent ceria hydroxy species are known to play an important role in the flotation of REM, particularly with hydroxamic acid, one of the most selective collectors of REM.^31,43^ However, the oxidation of trivalent Ce^III^ to Ce^IV^ during the storage of ores and tailings leads to the creation of a new surface chemistry on the particles with mixed Ce^III^/Ce^IV^ adsorption sites.^34−36^ In this study, cerium oxide particles with varying surface oxidation states were used as a model system to study the impact of redox properties on their interaction with the collector. We have found a clear correlation between the reduced state of CeO_2_^(red)^ ultrafine particles and their improved flotation performance with both biosurfactants and BHA compared to the oxidized CeO_2_^(ox)^ counterpart. In particular, BHA achieves a higher recovery for CeO_2_^(red)^ particles compared to CeO_2_^(ox)^ in single mineral flotation experiments (Figure 3b,c). Moreover, the selectivity of BHA is significantly higher for ultrafine reduced ceria than for oxidized ceria when floated against ultrafine hematite particles (Figures 4 and 5). In addition, ζ-potential measurements suggest that BHA can be adsorbed on CeO_2_^(red)^ through the surface precipitation of Ce-BHA complexes, while there is no signature of this reaction on CeO_2_^(ox)^. This finding supports the general mechanism of hydroxamic acid adsorption by trivalent Ce^III^ hydroxy species.

Similar to BHA, ASL prefers to interact with CeO_2_^(red)^ over CeO_2_^(ox)^. This stronger interaction at pH 4 is evidenced by the higher adsorption density of ASL on CeO_2_^(red)^ compared to that on CeO_2_^(ox)^ (Table 1). Furthermore, in single-mineral flotation experiments, ASL shows a higher recovery for CeO_2_^(red)^ than for CeO_2_^(ox)^ at pH 4 where its carboxyl group is mostly protonated (p*K*_a_ ca. 5).^63^ The ASL adsorption at pH 4 renders CeO_2_^(red)^ highly hydrophobic (Figure 8). We speculate that the adsorption mechanism of ASL involves the surface precipitation of a hydrophobic Ce^III^-ASL complex. This mechanism has earlier been postulated for the adsorption of deacetylated ASL on a copper sulfide.^30^ In contrast, ASL has a lower affinity for CeO_2_^(ox)^ as evidenced by single mineral flotation, adsorption density, and hydrophobicity tests. We hypothesize that with CeO_2_^(ox)^, ASL interacts directly with the Ce^IV^ surface sites, possibly resulting in a less hydrophobic conformation and slower adsorption kinetics. It follows from the low floatability of CeO_2_^(ox)^ at pH 4 and low adsorption densities even after the 2 h adsorption (Table 1). Further spectroscopic studies are needed to delineate the adsorption form of ASL on CeO_2_^(ox)^.

The ζ-potential results (Figure 7) suggest that LSL adsorbs on the metal oxides at acidic pH in the colloidal form. This process makes CeO_2_^(red)^ particles highly hydrophobic, inducing their hydrophobic aggregation/flocculation, which is mainly driven by the hydrophobic interactions between the ultrafine particles.^64^ Accordingly, no aggregate formation is observed for the moderately hydrophobic LSL-adsorbed hematite at pH 4 (Figure S7). As in the case of oleate flotation,^64−66^ the hydrophobic agglomeration in LSL flotation is likely to be promoted by the surfactant colloids acting as bridges between hydrophobic particles.

## 5 Conclusions

In the flotation of binary mineral mixtures without the use of depressants, the best collector of ultrafine ceria against hematite is BHA. In the three-mineral flotation, both ASL and LSL can separate reduced ceria and hematite as a group from quartz at pH 10. ASL can separate ultrafine reduced ceria from its mixture with ultrafine hematite and quartz at pH 7. LSL can separate hematite from reduced ceria and quartz at pH 4.

ASL, LSL, and BHA chemisorb on the metal oxides in the anionic form, which can be accompanied by surface precipitation. Surface precipitation is the dominant adsorption mechanism in the BHA-hematite system at pH 3–5 during prolonged (hours) conditioning times.

The fully oxidized state of ceria has a negative effect on its separation from hematite by all three collectors. This result agrees with the decrease in the affinity of the metal oxides to the biosurfactants at pH 4 in the order of α-Fe_2_O_3_ > CeO_2_^(red)^ > CeO_2_^(ox)^. The suppressive effect of full oxidation suggests that the adsorption sites of ceria include not only Ce^IV^–OH but also the frustrated Lewis pairs of Ce^III^ and oxygen vacancies.

The highest-grade-recovery figures in ceria flotation against hematite are achieved by BHA at pH 4. This result is explained by the hydroxylation of the surface Ce^IV^ cations at acidic pH, which is coupled with the slow kinetics of the BHA adsorption on hematite. Given that the optimum flotation pH ranges for bastnäsite and gangue metal oxides including hematite overlap at pH 9–10, this result suggests that, without employing depressants of gangue minerals, the partial oxidation of Ce-bearing REM can improve their separation by extending their optimum floatability range to acidic pH. The slow kinetics of BHA adsorption is behind the discrepancies between the trends observed in contact angle, ζ-potential, and single-mineral flotation.

The best separation of hematite from ceria is achieved by LSL for reduced ceria at pH 4. The 99% rejection of ceria particles is explained by their hydrophobic agglomeration to large, compact hydrophobic particles that act as a defoamer. The LSL colloids in the solution may play the role of bridges in the agglomeration.

Toward assessing the viability of the biosurfactants in real ore flotation, it is important to understand their interaction with a broader range of gangue minerals associated with REM, including calcite and aluminosilicates, and develop strategies to reject gangues by employing the flotation kinetics and depressants/regulators.

## Supporting Information Available

Materials characterization by surface tension, characterization of CeO_2_ particles, flocculation analysis, and XPS spectra of hematite and ceria particles (PDF)

## References

[ref1] Lèbre É.; Corder G.; Golev A. The Role of the Mining Industry in a Circular Economy: A Framework for Resource Management at the Mine Site Level. J. Ind. Ecol. 2017, 21 (3), 662–672. 10.1111/jiec.12596.

[ref2] Binnemans K.; Jones P. T.; Blanpain B.; Van Gerven T.; Pontikes Y. Towards zero-waste valorisation of rare-earth-containing industrial process residues: a critical review. J. Cleaner Prod. 2015, 99, 17–38. 10.1016/j.jclepro.2015.02.089.

[ref3] Marion C.; Li R.; Waters K. E. A review of reagents applied to rare-earth mineral flotation. Adv. Colloid Interface Sci. 2020, 279, 102142 10.1016/j.cis.2020.102142. 32244063

[ref4] Abaka-Wood G. B.; Ehrig K.; Addai-Mensah J.; Skinner W. Recovery of Rare Earth Elements Minerals from Iron-Oxide-Silicate-Rich Tailings: Research Review. Eng. 2022, 3 (2), 259–275. 10.3390/eng3020020.

[ref5] Chelgani S. C.; Rudolph M.; Leistner T.; Gutzmer J.; Peuker U. A. A review of rare earth minerals flotation: Monazite and xenotime. Int. J. Min. Sci. Technol. 2015, 25 (6), 877–883. 10.1016/j.ijmst.2015.09.002.

[ref6] Zhang J.; Edwards C. A review of rare earth mineral processing technology. In 44th annual meeting of the Canadian mineral processors. CIM, Ottawa, 2012, .79, p 102.

[ref7] Farrokhpay S.; Filippov L.; Fornasiero D. Flotation of Fine Particles: A Review. Miner. Process. Extr. Metall. Rev. 2020, 42, 473–483. 10.1080/08827508.2020.1793140.

[ref8] Sivamohan R. The problem of recovering very fine particles in mineral processing — A review. Int. J. Miner. Process. 1990, 28 (3–4), 247–288. 10.1016/0301-7516(90)90046-2.

[ref9] Fang J.; Ge Y.; Yu J. Effects of particle size and wettability on froth stability in a collophane flotation system. Powder Technol. 2021, 379, 576–584. 10.1016/j.powtec.2020.11.028.

[ref10] Johansson G.; Pugh R. J. The influence of particle size and hydrophobicity on the stability of mineralized froths. Int. J. Miner. Process. 1992, 34 (1–2), 1–21. 10.1016/0301-7516(92)90012-L.

[ref11] Abaka-Wood G. B.; Addai-Mensah J.; Skinner W. The Use of Mining Tailings as Analog of Rare Earth Elements Resources: Part 1 – Characterization and Preliminary Separation. Miner. Process. Extr. Metall. Rev. 2021, 43, 701–715. 10.1080/08827508.2021.1920410.

[ref12] Alcalde J.; Kelm U.; Vergara D. Historical assessment of metal recovery potential from old mine tailings: A study case for porphyry copper tailings, Chile. Miner. Eng. 2018, 127, 334–338. 10.1016/j.mineng.2018.04.022.

[ref13] Abaka-Wood G. B.; Zanin M.; Addai-Mensah J.; Skinner W. Recovery of rare earth elements minerals from iron oxide–silicate rich tailings – Part 1: Magnetic separation. Miner. Eng. 2019, 136, 50–61. 10.1016/j.mineng.2019.02.026.

[ref14] Silva C. M.; Lode S.; Aasly K.; Kowalczuk P. B. Early-stage application of process mineralogy methodologies for mineral tracking in flotation of rare earth elements (REE)-bearing minerals from a deposit in Norway. Miner. Eng. 2023, 202, 108268 10.1016/j.mineng.2023.108268.

[ref15] Schulz B.; Merker G.; Gutzmer J. Automated SEM Mineral Liberation Analysis (MLA) with Generically Labelled EDX Spectra in the Mineral Processing of Rare Earth Element Ores. Minerals 2019, 9 (9), 527 10.3390/min9090527.

[ref16] Beard C. D.; Goodenough K. M.; Borst A. M.; Wall F.; Siegfried P. R.; Deady E. A.; Pohl C.; Hutchison W.; Finch A. A.; Walter B. F.; et al. Alkaline-Silicate REE-HFSE Systems. Econ. Geol. 2023, 118 (1), 177–208. 10.5382/econgeo.4956.

[ref17] Julapong P.; Numprasanthai A.; Tangwattananukul L.; Juntarasakul O.; Srichonphaisarn P.; Aikawa K.; Park I.; Ito M.; Tabelin C. B.; Phengsaart T. Rare Earth Elements Recovery from Primary and Secondary Resources Using Flotation: A Systematic Review. Appl. Sci. 2023, 13 (14), 8364 10.3390/app13148364.

[ref18] Keth J.; Johann T.; Frey H. Hydroxamic Acid: An Underrated Moiety? Marrying Bioinorganic Chemistry and Polymer Science. Biomacromolecules 2020, 21 (7), 2546–2556. 10.1021/acs.biomac.0c00449. 32525665

[ref19] Pang W.; Yao J.; Knudsen T. S. ˇ.; Cao Y.; Liu B.; Li H.; Li M.; Zhu J. Degradation of three typical hydroxamic acids collectors via UVA-B activated H2O2 and persulfate: Kinetics, transformation pathway, DFT calculation and toxicity evaluation. Chem. Eng. J. 2023, 451, 138639 10.1016/j.cej.2022.138639.

[ref20] Jain G.; Havskjold H.; Dhar P.; Ertesvåg H.; Chernyshova I.; Kota H. R. Green Foam-Based Methods of Mineral and Ion Separation. In Multidisciplinary Advances in Efficient Separation Processes; Chernyshova I., Ponnurangam S., Liu Q., Eds.; American Chemical Society, 2020; Vol. 1348, pp 265–301. 10.1021/bk-2020-1348.ch009.

[ref21] Chernyshova I.; Slabov V.; Kota H. R. Emerging application of biosurfactants in metal extraction. Curr. Opin. Colloid Interface Sci. 2023, 68, 101763 10.1016/j.cocis.2023.101763.

[ref22] Díaz De Rienzo M.; Kamalanathan I. D.; Martin P. J. Comparative study of the production of rhamnolipid biosurfactants by B. thailandensis E264 and P. aeruginosa ATCC 9027 using foam fractionation. Process Biochem. 2016, 51 (7), 820–827. 10.1016/j.procbio.2016.04.007.

[ref23] Hu X.; Subramanian K.; Wang H.; Roelants S. L. K. W.; To M. H.; Soetaert W.; Kaur G.; Lin C. S. K.; Chopra S. S. Guiding environmental sustainability of emerging bioconversion technology for waste-derived sophorolipid production by adopting a dynamic life cycle assessment (dLCA) approach. Environ. Pollut. 2021, 269, 116101 10.1016/j.envpol.2020.116101. 33307395

[ref24] Hu X.; Subramanian K.; Wang H.; Roelants S. L. K. W.; Soetaert W.; Kaur G.; Lin C. S. K.; Chopra S. S. Bioconversion of Food Waste to produce Industrial-scale Sophorolipid Syrup and Crystals: dynamic Life Cycle Assessment (dLCA) of Emerging Biotechnologies. Bioresour. Technol. 2021, 337, 125474 10.1016/j.biortech.2021.125474. 34320754

[ref25] Hogan D. E.; Curry J. E.; Pemberton J. E.; Maier R. M. Rhamnolipid biosurfactant complexation of rare earth elements. J. Hazard. Mater. 2017, 340, 171–178. 10.1016/j.jhazmat.2017.06.056. 28715740

[ref26] Tang J.; He J.; Qiu Z.; Xin X. Metal removal effectiveness, fractions, and binding intensity in the sludge during the multiple washing steps using the combined rhamnolipid and saponin. J. Soils Sediments 2019, 19 (3), 1286–1296. 10.1007/s11368-018-2106-0.

[ref27] Zhou D.; Li Z.; Luo X.; Su J. Leaching of rare earth elements from contaminated soils using saponin and rhamnolipid bio-surfactant. J. Rare Earths 2017, 35 (9), 911–919. 10.1016/S1002-0721(17)60994-3.

[ref28] Slabov V.; Jain G.; Larsen E.; Kota H. R.; Chernyshova I. Eco-Friendly Collectors for Flotation of Fine Hematite and Malachite Particles. Min., Metall., Explor. 2023, 40 (2), 475–492. 10.1007/s42461-023-00743-z.

[ref29] Dhar P.; Thornhill M.; Roelants S.; Soetaert W.; Chernyshova I. V.; Rao Kota H. Linking molecular structures of yeast-derived biosurfactants with their foaming, interfacial, and flotation properties. Miner. Eng. 2021, 174, 107270 10.1016/j.mineng.2021.107270.

[ref30] Dhar P.; Havskjold H.; Thornhill M.; Roelants S.; Soetaert W.; Kota H. R.; Chernyshova I. Toward green flotation: Interaction of a sophorolipid biosurfactant with a copper sulfide. J. Colloid Interface Sci. 2021, 585, 386–399. 10.1016/j.jcis.2020.11.079. 33307307

[ref31] Pradip; Fuerstenau D. W. The adsorption of hydroxamate on semi-soluble minerals. Part I: Adsorption on Barite, Calcite and Bastnaesite. Colloids Surf. 1983, 8 (2), 103–119. 10.1016/0166-6622(83)80079-1.

[ref32] Massari S.; Ruberti M. Rare earth elements as critical raw materials: Focus on international markets and future strategies. Resour. Policy 2013, 38 (1), 36–43. 10.1016/j.resourpol.2012.07.001.

[ref33] Schüler D.; Buchert M.; Liu R.; Dittrich S.; Merz C. Study on rare earths and their recycling; Öko-Institut eV Darmstadt, 2011; Vol. 49, pp 30–40.

[ref34] Cui J.; Hope G. A.; Buckley A. N. Spectroscopic investigation of the interaction of hydroxamate with bastnaesite (cerium) and rare earth oxides. Miner. Eng. 2012, 36–38, 91–99. 10.1016/j.mineng.2012.03.001.

[ref35] Bolanz R. M.; Kiefer S.; Göttlicher J.; Steininger R. Hematite (α-Fe2O3) – A potential Ce4+ carrier in red mud. Sci. Total Environ. 2018, 622–623, 849–860. 10.1016/j.scitotenv.2017.12.043. 29227935

[ref36] Mullins D. R. The surface chemistry of cerium oxide. Surf. Sci. Rep. 2015, 70 (1), 42–85. 10.1016/j.surfrep.2014.12.001.

[ref37] Zhang S.; Huang Z.-Q.; Ma Y.; Gao W.; Li J.; Cao F.; Li L.; Chang C.-R.; Qu Y. Solid frustrated-Lewis-pair catalysts constructed by regulations on surface defects of porous nanorods of CeO2. Nat. Commun. 2017, 8 (1), 15266 10.1038/ncomms15266. 28516952 PMC5454379

[ref38] Chen H.-I.; Chang H.-Y. Synthesis of nanocrystalline cerium oxide particles by the precipitation method. Ceram. Int. 2005, 31 (6), 795–802. 10.1016/j.ceramint.2004.09.006.

[ref39] Lee H. B.; Bogart J. A.; Carroll P. J.; Schelter E. J. Structural and electrochemical characterization of a cerium(iv) hydroxamate complex: implications for the beneficiation of light rare earth ores. Chem. Commun. 2014, 50 (40), 5361–5363. 10.1039/C3CC46486E. 24177048

[ref40] Nakhaei F.; Irannajad M. Reagents types in flotation of iron oxide minerals: A review. Miner. Process. Extr. Metall. Rev. 2018, 39 (2), 89–124. 10.1080/08827508.2017.1391245.

[ref41] Zhang W. C.; Honaker R. A fundamental study of octanohydroxamic acid adsorption on monazite surfaces. Int. J. Miner. Process. 2017, 164, 26–36. 10.1016/j.minpro.2017.05.006.

[ref42] Wanhala A. K.; Doughty B.; Bryantsev V. S.; Wu L.; Mahurin S. M.; Jansone-Popova S.; Cheshire M. C.; Navrotsky A.; Stack A. G. Adsorption mechanism of alkyl hydroxamic acid onto bastnäsite: Fundamental steps toward rational collector design for rare earth elements. J. Colloid Interface Sci. 2019, 553, 210–219. 10.1016/j.jcis.2019.06.025. 31203005

[ref43] Sarvaramini A.; Azizi D.; Larachi F. Hydroxamic acid interactions with solvated cerium hydroxides in the flotation of monazite and bastnäsite—Experiments and DFT study. Appl. Surf. Sci. 2016, 387, 986–995. 10.1016/j.apsusc.2016.07.044.

[ref44] Raghavan S.; Fuerstenau D. W. The adsorption of aqueous octylhydroxamate on ferric oxide. J. Colloid Interface Sci. 1975, 50 (2), 319–330. 10.1016/0021-9797(75)90235-0.

[ref45] Fuerstenau D. W.; Pradip Zeta potentials in the flotation of oxide and silicate minerals. Adv. Colloid Interface Sci. 2005, 114–115, 9–26. 10.1016/j.cis.2004.08.006. 16007737

[ref46] Holder C. F.; Schaak R. E. Tutorial on Powder X-ray Diffraction for Characterizing Nanoscale Materials. ACS Nano 2019, 13 (7), 7359–7365. 10.1021/acsnano.9b05157. 31336433

[ref47] Scholes F. H.; Hughes A. E.; Hardin S. G.; Lynch P.; Miller P. R. Influence of Hydrogen Peroxide in the Preparation of Nanocrystalline Ceria. Chem. Mater. 2007, 19 (9), 2321–2328. 10.1021/cm063024z.

[ref48] Miller J. D.; Fuerstenau M. C.; Harper R. W. Hydroxamate vs. fatty acid flotation of iron oxide. Transactions. SME/AIME 1970, 247, 69–73.

[ref49] Jordens A.; Marion C.; Kuzmina O.; Waters K. E. Surface chemistry considerations in the flotation of bastnäsite. Miner. Eng. 2014, 66–68, 119–129. 10.1016/j.mineng.2014.04.013.

[ref50] Hayes S. A.; Yu P.; O’Keefe T. J.; O’Keefe M. J.; Stoffer J. O. The Phase Stability of Cerium Species in Aqueous Systems: I. E-pH Diagram for the System. J. Electrochem. Soc. 2002, 149 (12), C623 10.1149/1.1516775.

[ref51] Bouchaud B.; Balmain J.; Bonnet G.; Pedraza F. pH-distribution of cerium species in aqueous systems. J. Rare Earths 2012, 30 (6), 559–562. 10.1016/S1002-0721(12)60091-X.

[ref52] Sanghavi S.; Wang W.; Nandasiri M. I.; Karakoti A. S.; Wang W.; Yang P.; Thevuthasan S. Investigation of trimethylacetic acid adsorption on stoichiometric and oxygen-deficient CeO2(111) surfaces. Phys. Chem. Chem. Phys. 2016, 18 (23), 15625–15631. 10.1039/C6CP00855K. 27220740

[ref53] Ji P.; Wang L.; Chen F.; Zhang J. Ce3+-Centric Organic Pollutant Elimination by CeO2 in the Presence of H2O2. ChemCatChem 2010, 2 (12), 1552–1554. 10.1002/cctc.201000191.

[ref54] Wang W.; Thevuthasan S.; Wang W.; Yang P. Theoretical Study of Trimethylacetic Acid Adsorption on CeO2(111) Surface. J. Phys. Chem. C 2016, 120 (5), 2655–2666. 10.1021/acs.jpcc.5b09790. 27220740

[ref55] Hunter R. J.. In Chapter 8 - Influence of More Complex Adsorbates on Zeta Potential. In Zeta Potential in Colloid Science; Hunter R. J., Ed.; Academic Press, 1981; pp 305–344.

[ref56] Ponnurangam S.; Chernyshova I. V.; Somasundaran P. Rational Design of Interfacial Properties of Ferric (Hydr)oxide Nanoparticles by Adsorption of Fatty Acids from Aqueous Solutions. Langmuir 2012, 28 (29), 10661–10671. 10.1021/la300995g. 22694303

[ref57] Kosmulski M. Isoelectric points and points of zero charge of metal (hydr)oxides: 50years after Parks’ review. Adv. Colloid Interface Sci. 2016, 238, 1–61. 10.1016/j.cis.2016.10.005. 27890403

[ref58] Nabavi M.; Spalla O.; Cabane B. Surface chemistry of nanometric ceria particles in aqueous dispersions. J. Colloid Interface Sci. 1993, 160 (2), 459–471. 10.1006/jcis.1993.1417.

[ref59] De Faria L. A.; Trasatti S. The point of zero charge of CeO2. J. Colloid Interface Sci. 1994, 167 (2), 352–357. 10.1006/jcis.1994.1370.

[ref60] Vincent A.; Inerbaev T. M.; Babu S.; Karakoti A. S.; Self W. T.; Masunov A. E.; Seal S. Tuning Hydrated Nanoceria Surfaces: Experimental/Theoretical Investigations of Ion Exchange and Implications in Organic and Inorganic Interactions. Langmuir 2010, 26 (10), 7188–7198. 10.1021/la904285g. 20131920 PMC2876981

[ref61] Tam J.; Feng B.; Ikuhara Y.; Ohta H.; Erb U. Crystallographic orientation–surface energy–wetting property relationships of rare earth oxides. J. Mater. Chem. A 2018, 6 (38), 18384–18388. 10.1039/C8TA04938F.

[ref62] Kujawa M.; Volc J.; Halada P.; Sedmera P.; Divne C.; Sygmund C.; Leitner C.; Peterbauer C.; Haltrich D. Properties of pyranose dehydrogenase purified from the litter-degrading fungus Agaricus xanthoderma. FEBS J. 2007, 274 (3), 879–894. 10.1111/j.1742-4658.2007.05634.x. 17227387

[ref63] Laskowski J. S. Electrokinetic Measurements in Aqueous Solutions of Weak Electrolyte Type Surfactants. J. Colloid Interface Sci. 1993, 159 (2), 349–353. 10.1006/jcis.1993.1333.

[ref64] Song S.; Lu S. Hydrophobic Flocculation of Fine Hematite, Siderite, and Rhodochrosite Particles in Aqueous Solution. J. Colloid Interface Sci. 1994, 166 (1), 35–42. 10.1006/jcis.1994.1268.

[ref65] Kulkarni R. D.; Somasundaran P. Flotation chemistry of hematite/oleate system. Colloids Surf. 1980, 1 (3–4), 387–405. 10.1016/0166-6622(80)80025-4.

[ref66] Akdemir U. ¨.; Hiçyilmaz C. Shear flocculation of chromite fines in sodium oleate solutions. Colloids Surf A. 1996, 110 (1), 87–93. 10.1016/0927-7757(95)03428-5.

