# Supplementary material for: Impact of the Ceria Particle Oxidation State on the Collecting Properties of Sophorolipids and Benzohydroxamic Acid
Source: ACS Omega. 2024 Sep 27;9(40):41456–67. doi: 10.1021/acsomega.4c04818 (PMC11465557; doi:10.1021/acsomega.4c04818)
Supplement: Supplementary file 1 — ao4c04818_si_001.pdf [file ao4c04818_si_001.pdf]

Supplementary information

The impact of ceria particle oxidation state on the collecting properties of sophorolipids and benzohydroxamic acid

Vladislav Slabov[a]*, Hanumantha Rao Kota[a], and Irina Chernyshova[a,b]

[a]*Department of Geoscience and Petroleum, Norwegian University of Science and Technology (NTNU), NO-7031, Trondheim, Norway*

[b]*Department of Earth and Environmental Engineering, Columbia University, New York, NY 10027, USA*

Table of content

S1. Surface tension..........2

S2. Surface excess (Γ_{max}) and minimum surface area (A_{min}) of ASL and LSL 3

S3. Characterization of ultrafine metal oxide particles 3

S4. ζ-potential of $CeO_2^{(ox)}$ particles before and after cleaning in HNO_3 6

S5. Flocculation during Flotation of $CeO_2^{(red)}$ and α-Fe_2O_3 with 100 µM LSL at pH 4..........7

S6. Hydrophobicity test using high speed camera.......... 10

S7. Effect of oxidation of $CeO_2^{(red)}$ on its flotation with ASL and LSL.......... 10

S8. XPS of initial and acid-washed hematite.......... 13

References 14

S1. Surface tension

Static surface tension was measured by Du Nouy's ring method using a Biolin Scientific Sigma 702 instrument. The ring was repeatedly flamed until it glowed red-hot in ethanol flame. Afterward, it was washed with deionized water to ensure the complete removal of impurities. The instrument was calibrated with water (72 ± 1 mN/m). The stock solutions of LSL or ASL were adjusted to pH 4, and then added to water with the same pH. The pH of the final solution did not drift by more than 0.1 pH. Each reported surface tension data point is an average of 5 measurements in the same solution. Each set of experiments was repeated on two solutions at specific pH and concentration. Differences between the duplicates were insignificant.

As shown in Figure S1, the surface tension of ASL is pH dependent, while the effect of pH is different in different concentration ranges. The critical micelle concentration (CMC) of ASL extracted from the surface tension curve at pH 4 is 110 µM. CMC increases at pH 6 and pH 10 to 140 µM and 180 µM, respectively, which can be related to the ionization of the carboxylic group of ASL [1].

LSL has higher interfacial activity at pH 4 compared to ASL (Figure S1b). Its CMC is 70 µM. Taking into account that ASL is also mostly non-ionic (protonated) at this pH (pK_a of oleic acid is 5), the higher interfacial activity of LSL can be explained by its more rigid structure compared to the sterically flexible bola structure of ASL (Figure S1a). The diacetylated LSL used in our study is less surface active compared to earlier studied diacetylated and monoacetylated LSL, which have CMC of 20 mg/ L (29 µM) and 15 mg/L (22 µM) at pH 7.4, respectively [2]. This discrepancy can tentatively be explained by the colloidal form of LSL in the solution [1], combined with the partial base hydrolysis of LSL (saponification of its ester bonds) in the stock solution at pH 10 [3]. At pH 7, the surface tension of LSL increases and remains practically the same at pH 10. As this effect is untypical of non-ionic surfactants, it can tentatively be ascribed to the partial hydrolysis of LSL.

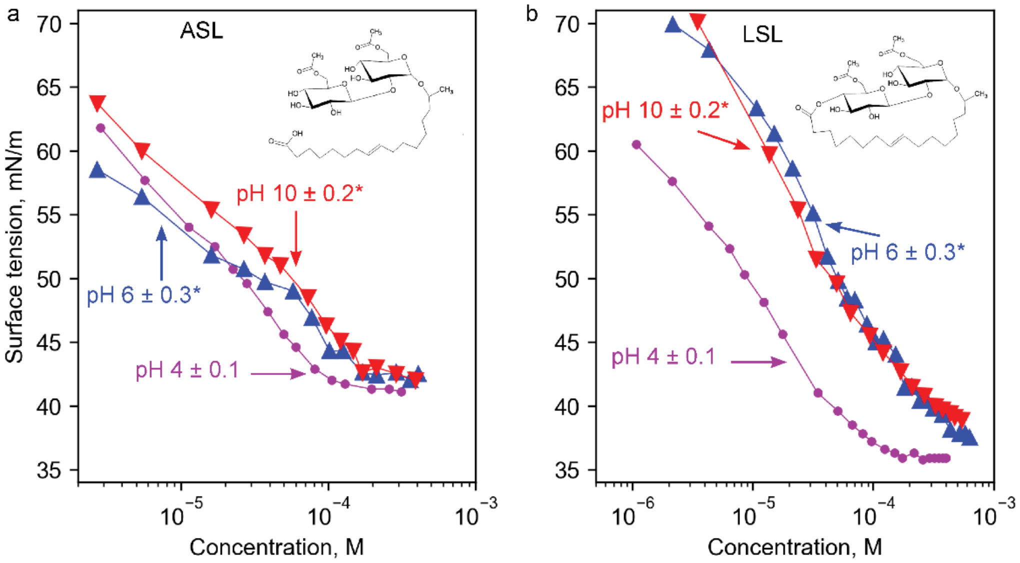

Figure S1 Surface tension of (a) ASL, and (b) LSL at pH 4 ± 0.1. The data at pH 6 ± 0.3 and 10 ± 0.2 are from our precious study [1].

S2. Surface excess (Γ_{max}) and minimum surface area (A_{min}) of ASL and LSL

The minimum surface area per molecule is used to convert the TOC data into the number of adsorbed monolayers of the biosurfactants. The surface tension (γ) values of ASL and LSL at pH 4 were used to determine the surface excess (Γ_{max}) and minimum surface area (A_{min}) from the slope of the linear part of γ vs ln C using the following equations,[4]

$$\Gamma_{max} = -\frac{1}{nRT} \times \frac{d\gamma}{dlnC},$$

$$A_{min} = \frac{1}{N\Gamma_{max}},$$

Where R is the gas constant (8.314 $J \cdot K^{-1} \cdot mol^{-1}$), N is the Avogadro's number, and n is the molecule specific dissociation number (the Gibbs prefactor). We used Gibbs prefactor n = 1. The A_{min} values for ASL and LSL at pH 4 are 88 $Å^2$/ molecule and 94 $Å^2$/ molecule, respectively.

S3. Characterization of ultrafine metal oxide particles

S3.1. Purity, crystallinity, morphology, particle size, and BET surface area of ultrafine metal oxide particles

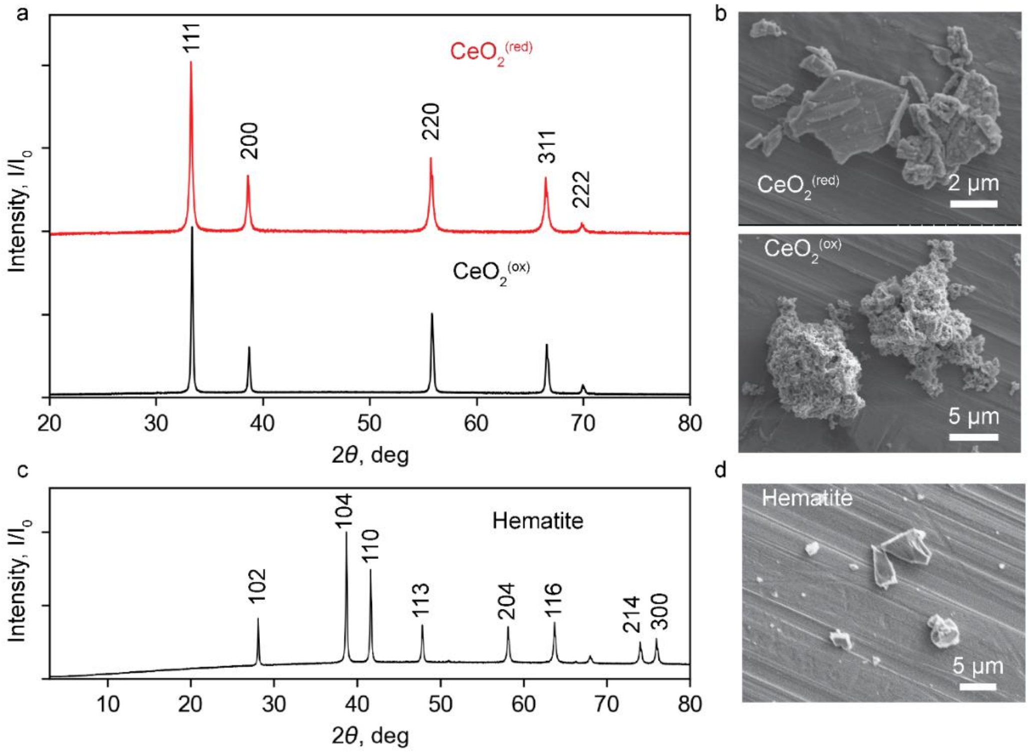

Figure S2 – (a) XRD of $CeO_2^{(red)}$ (red line), $CeO_2^{(ox)}$ (black line); (b) SEM images of $CeO_2^{(red)}$ (top) and $CeO_2^{(ox)}$ (bottom) particles, (c) XRD and (d) SEM images of α-Fe_2O_3. The particles have purity > 99 %.

Hematite particles are 99% phase pure (Figure S2a), while XPS detects only traces of Si (Figure S12a). The shapes of the hematite particles are irregular, with sharp edges (Figure 3b). The BET surface area is 2.1 m^2/g. The D_{90}, D_{50}, and D_{10} particle size distribution is 12.3, 3.7, and 0.8 μm, respectively (Table 1).

XPS does not detect any foreign element on the ceria surface except for adventitious carbon (Figure 4a). XRD confirms that both $CeO_2^{(red)}$ and $CeO_2^{(ox)}$ have a polycrystalline cubic ceria structure and do not contain other phases (Figure S2b) [5]. The larger average crystallite size of $CeO_2^{(ox)}$ compared to $CeO_2^{(red)}$ (530 vs. 430 nm) indicates that the former is more crystalline (Table S1). $CeO_2^{(red)}$ and $CeO_2^{(ox)}$ particles have preferable orientation along the (111) crystallographic plane, which is the most stable and the least reactive compared to (001) and (110) [6]. Notably, $CeO_2^{(red)}$ has a higher fraction of the CeO_2(220) and (200) facets (Table 1), which are more catalytically active compared to (111) [7].

$CeO_2^{(ox)}$ has a higher BET surface area (12.8 m^2/g) and lower (19 ± 2 μm) particle size compared to $CeO_2^{(red)}$ (10.5 m^2/g and 27 ± 2 μm, respectively). Accordingly, SEM shows that $CeO_2^{(ox)}$ has a highly porous surface, in contrast to $CeO_2^{(red)}$ (Figure S2b).

In summary, even though the particle size of the reduced and oxidized ceria particles is larger by a factor of 1.5-2 than that of hematite, their surface area is larger by a factor 5-6, which can be explained by their higher surface roughness (Figure S2bd).

Table S1 - BET surface area, particle size distribution and crystallite size of $CeO_2^{(red)}$, $CeO_2^{(ox)}$ and hematite particles. $I_{111/200}$ and $I_{111/220}$ are the relative intensities of the XRD signal from the (111) crystallographic plane to that from the (200) and (220) planes, respectively. The crystallite size was determined using the Scherrer equation.

Sample	Surface area (BET), m^2/g	Particle size distribution, μm			Crystallite size (D_{111}), nm	I_{111}/I_{200}	I_{111}/I_{220}
		d_{90}	d_{50}	d_{10}			
$CeO_2^{(red)}$	10.5 ± 0.2	27 ± 2	11.0 ± 0.9	1.6 ± 0.1	430	2.6	2.0
$CeO_2^{(ox)}$	12.8 ± 0.3	19 ± 2	4.6 ± 0.4	0.6 ± 0.2	530	3.2	2.0
α-Fe_2O_3	2.1 ± 0.2	12 ± 1	3.7 ± 0.4	0.8 ± 0.2	-	-	-

S3.2. The oxidation state of ceria particles

XPS confirms that the surface of $CeO_2^{(red)}$ is more reduced compared to that of $CeO_2^{(ox)}$. The former is characterized by more pronounced shoulders of Ce^{III} at 885 eV and 904 eV in the Ce 3d spectra (Figure S3b) [8]. A higher relative concentration of Ce^{III} on the $CeO_2^{(red)}$ surface is also evidenced by the lower relative intensity of the Ce 3d peak at 917 eV which is observed only for stoichiometric CeO_2 [8]. The more reduced state of the $CeO_2^{(red)}$ particles is also evidenced by their yellowish color while the $CeO_2^{(ox)}$ particles are almost white (Figure S7a,b). The yellowing color of non-stochiometric ceria is explained by the decrease in the ceria bandgap by Ce^{III} [9].

The O 1s spectra show that, compared to $CeO_2^{(ox)}$, $CeO_2^{(red)}$ has a more hydroxylated surface and its OH groups are more basic. The main peak at 529.3 eV in the O 1s spectra is due to oxygen in the CeO_2 lattice [10] (Figure S3c). The weaker peaks at 531.2 and 531.8 eV are assigned to the surface OH groups of $CeO_2^{(red)}$ and $CeO_2^{(ox)}$, respectively [10, 11]. The twice higher relative intensity of the OH peak of $CeO_2^{(red)}$ indicates that the $CeO_2^{(red)}$ surface is twice more hydroxylated compared to $CeO_2^{(ox)}$. The lower binding energy of the OH peak of $CeO_2^{(red)}$ indicates that surface hydroxyls of $CeO_2^{(red)}$ on average are more basic, which can be linked to the presence of less acidic Ce^{III} cations in their vicinity. Finally, the C 1s spectra of the ceria particles contain the main C 1s peak at 285.0 eV, which is due to the sp^3 carbon of carbonaceous contaminations, as well as a minor peak at 289 eV (Figure S3d). The latter overlaps with the Ce 4s peak [12] and hence cannot be assigned unambiguously.

In the Raman spectra, $CeO_2^{(red)}$ is characterized by a blue shift and broadening of the F_{2g} cubic fluorite CeO_2 Raman peak at 465 cm^{-1} (Figure S3e) which is typical of the reduced ceria state [13]. The absence of the typical peak of the oxygen vacancies at 600 cm^{-1} in the spectrum of $CeO_2^{(red)}$ is explained by the low specific surface area of the particles.

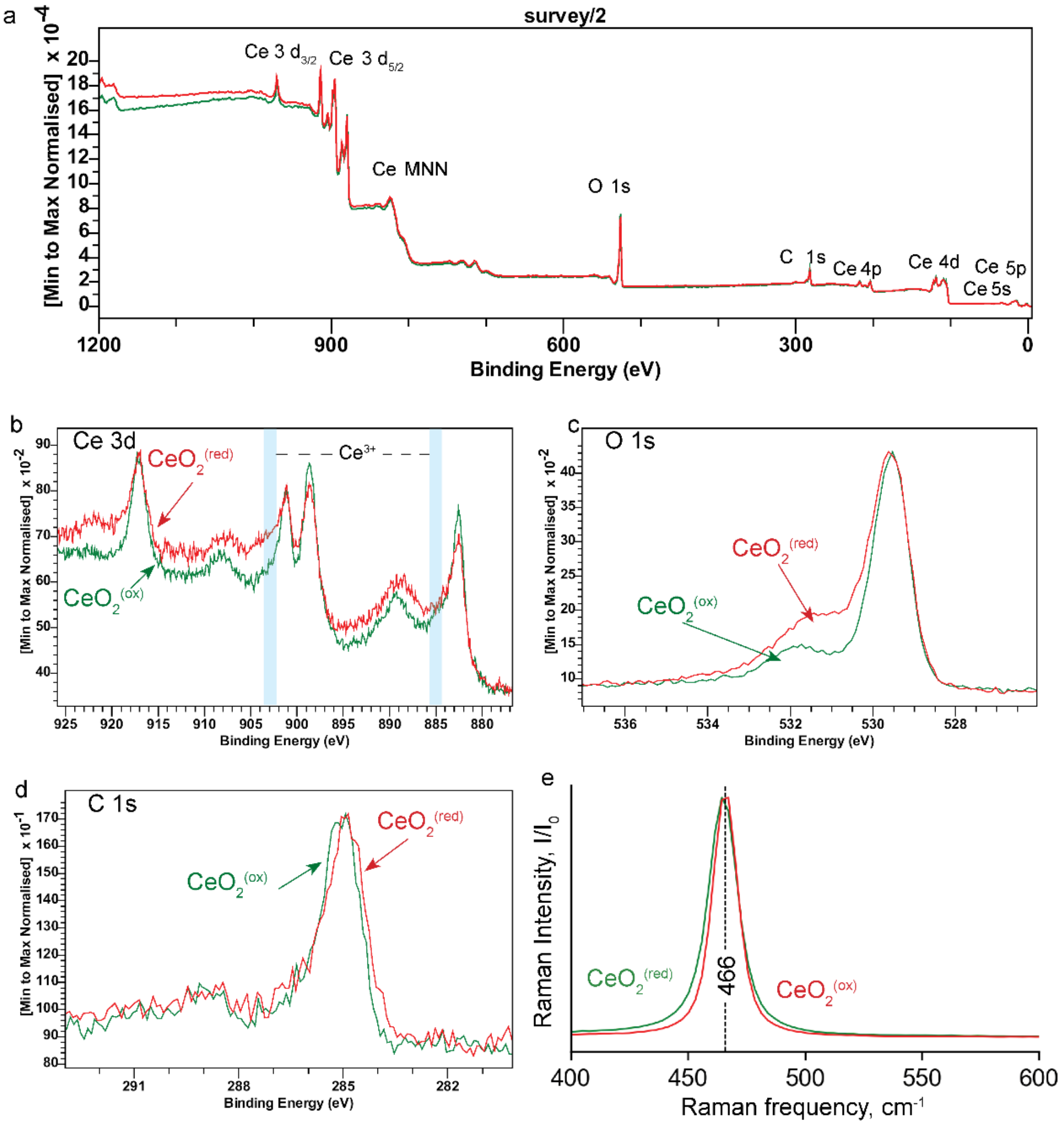

Figure S3 (a,b,c,d) XPS and (e) Raman spectra of $CeO_2^{(red)}$ – green line, $CeO_2^{(ox)}$ – red line: (a) survey spectra, (b) Ce 3d, (c) O 1s, and (d) C 1s spectra. The vertical blue bars in (b) mark shoulders caused by Ce^{III}. Peak assignment is based on Refs [8, 10].

The CeO_2 nanoparticles used for the synthesis of CeO_2^{ox} were analyzed by transmission electron microscopy (TEM) and zeta potential. For the TEM analysis, the nanoparticles were sonicated for 30 min in ethanol to obtain very diluted dispersion, which further was drop-cast onto TEM copper grid and dried out. TEM images were acquired using a JEOL 2100F electron microscope operated at 200 kV. The sample preparation for zeta potential measurements is described in the main text.

As seen from the TEM images (Figure S4a), nanoceria particles have a random, almost cubic shape. Its electron diffraction pattern of confirms the random orientation of nanoparticles (Figure S4b).

Zeta (ζ) potential on Figure S2c shows that nanoceria has isoelectric point (IEP) is ca pH 7. This value is close to the values of 8 previously reported for cerium oxide nanoparticles.[14]

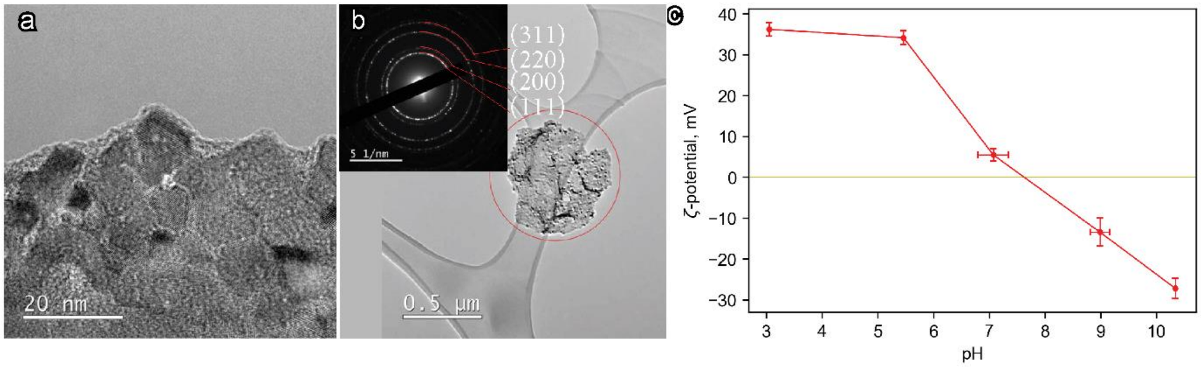

Figure S4 Characterization of CeO_2 nanoparticles used for preparation of $CeO_2^{(ox)}$ microparticles: (a) and (b) transmission electron microscopy (TEM) analysis, (c) ζ-potential of CeO_2 nanoparticles.

S4. ζ-potential of $CeO_2^{(ox)}$ particles before and after cleaning in HNO_3

ζ-potential of unwashed and acid washed $CeO_2^{(ox)}$ particles were measured to exclude the effect of organic contaminations as a possible reason of the difference in the IEP of $CeO_2^{(ox)}$ and $CeO_2^{(red)}$ (Figure S5).

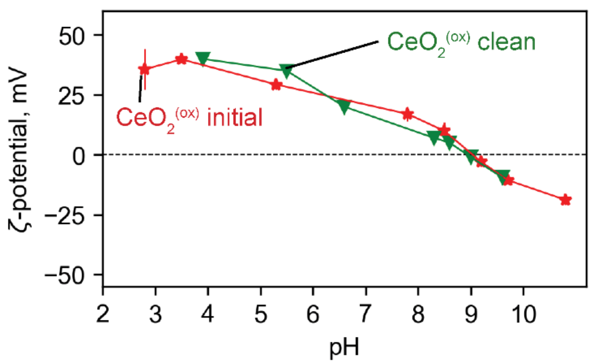

Figure S5 ζ-potential of $CeO_2^{(ox)}$ particles before and after cleaning in a 0.001 M HNO_3 solution for overnight. The total organic carbon of the solutions from the filtered dispersion has values of 70 mg/L for the initial particles and 2 mg/L for the clean particles.

S5. Flocculation during Flotation of $CeO_2^{(red)}$ and α-Fe_2O_3 with 100 µM LSL at pH 4

$CeO_2^{(red)}$ is not floatable with 100 µM LSL at pH 4 (Figure 5), while the froth which is unstable (Figure S6a, and video link [https://youtu.be/zQf928n_s04]). In contrast, the recovery is as high as 97% and the froth is strong in the flotation of hematite under the same conditions (Figure S6b and video link[https://youtu.be/zQf928n_s04]). During the flotation of the $CeO_2^{(red)}$-α-Fe_2O_3 binary system, the froth has an intermediate strength (Figure S6c).

The weak froth formed during the $CeO_2^{(red)}$ flotation can be explained by the defoaming effect of the ~100 µm hydrophobic $CeO_2^{(red)}$ flocs formed by the ultrafine particles in the pH 4 LSL solution. The high hydrophobicity of $CeO_2^{(red)}$ conditioned in the LSL solution at pH 4 is reported in *section 3.2.3* of the main text. The formation of the large flocs is shown in Figure S7b (see below for more detail).

To understand why hydrophobic $CeO_2^{(red)}$ is not floated in contrast to less hydrophobic hematite, we compared the flotation of $CeO_2^{(red)}$ at pH 4 with LSL, LSL and a frother (Dowfroth 200), and LSL-ASL mixtures, along with the structures of the corresponding froths and the agglomeration of the particles in the tailings. The LSL and LSL+ASL concentration was 100 µM. The frother concentration was ca 50 µM. In the mixtures, the LSL:ASL ratios were 9:1 and 7:3. As a reference, $CeO_2^{(red)}$ was floated with the frother in the absence of a biosurfactants, as well as with 30 µM ASL without a frother. The froth was analyzed visually directly in the flotation cell and in a Petri dish under an optical microscope.

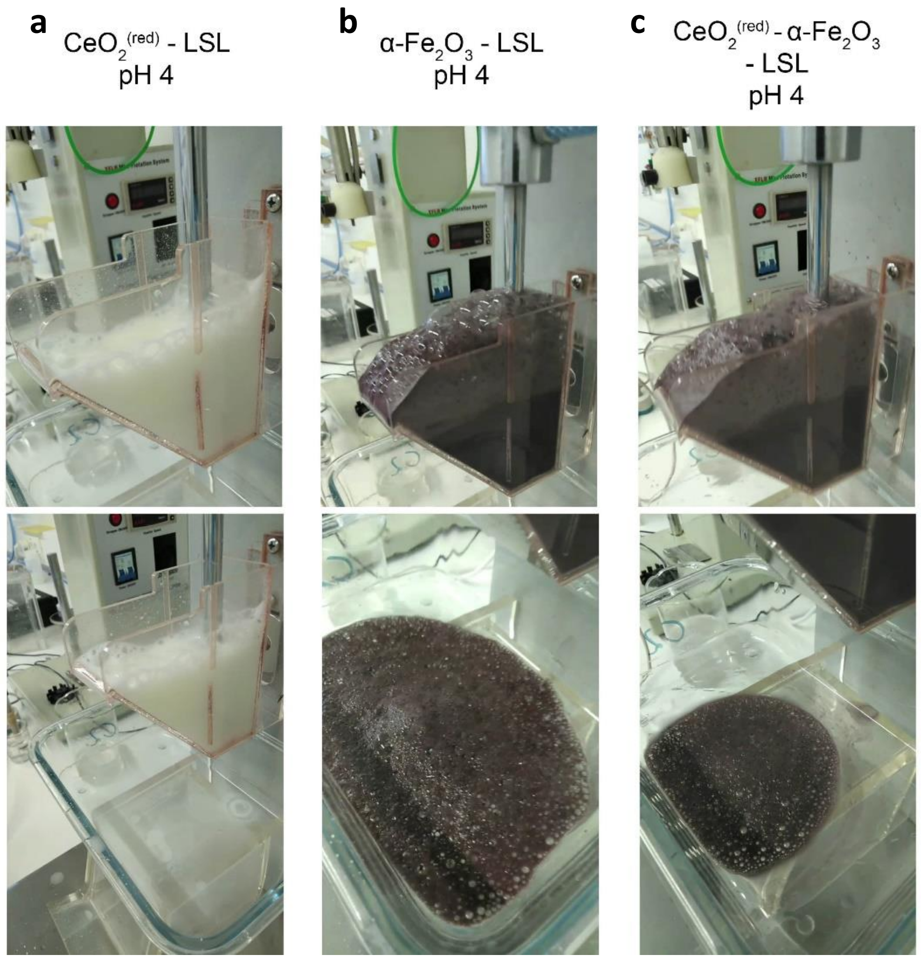

Figure S6 Flotation of ultrafine (<20 µm) (a) $CeO_2^{(red)}$ (b) α-Fe_2O_3, and (c) their 1:1 mixture with 100 µM LSL at pH 4. Video link [https://youtu.be/zQf928n_s04]

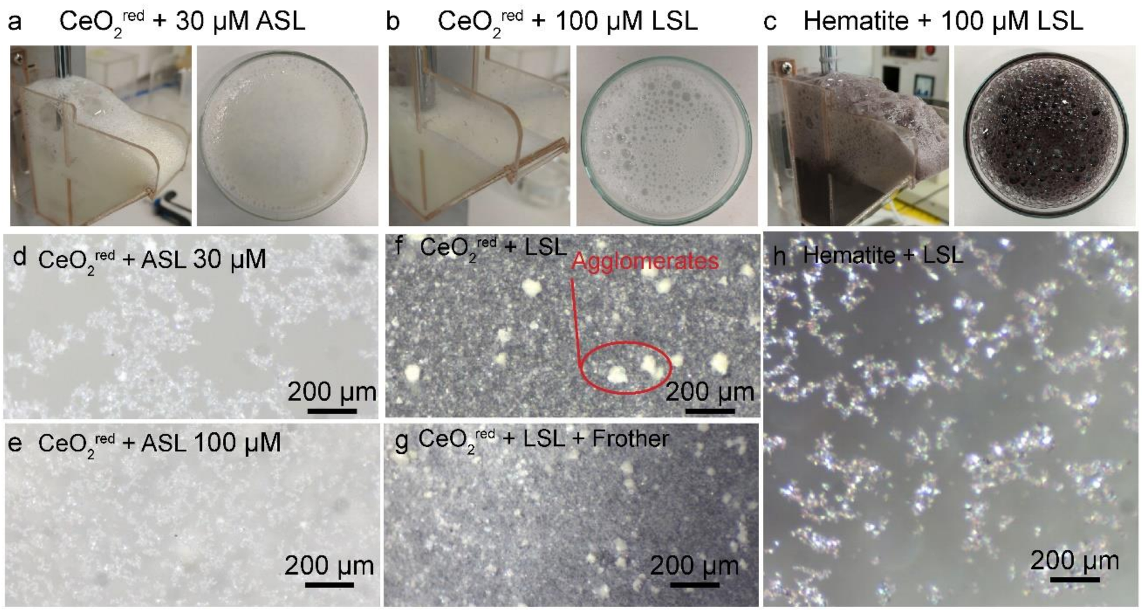

Figure S7 Visual analysis of the flotation of (a) $CeO_2^{(red)}$ with 30 µM ASL, (b) $CeO_2^{(red)}$ with 100 µM LSL, and (c) hematite with 100 µM LSL. On the top photos of froth in the flotation cell and collected in the Petri dish.

In the collectorless flotation of $CeO_2^{(red)}$ using Dowfroth 200, a noticeable froth is produced. However, the recovery is only 10%, indicating that the natural hydrophobicity of $CeO_2^{(red)}$ is not high enough (*section 3.2.3*). The flotation with 30 µM and 100 µM ASL recovers ~50% and ~90% $CeO_2^{(red)}$, respectively, showing a good froth in both cases (Figure S7a). The hydrophobic layer of $CeO_2^{(red)}$ particles resting on the water consists of particles arranged in loosely structured fractal patterns that continuously shift due to the Brownian motion (Figure S7d,e). Apparently, these particles avoid extensive agglomeration and remain predominantly dispersed.

However, when $CeO_2^{(red)}$ is floated with LSL, the froth is weak and unstable, containing agglomerates with a diameter of 50-100 µm (Figure S7b, e). The addition of the frother does improves neither the LSL flotation nor the froth, while the same agglomerates were formed (Figure S7f). Given that the LSL-adsorbed $CeO_2^{(red)}$ is highly hydrophobic (Figure 4b), these results suggest that the defoaming effect and thereby the low floatability of $CeO_2^{(red)}$ at pH 4 are caused by the large hydrophobic agglomerates.

Notably, the same low recovery of $CeO_2^{(red)}$ and the same unstable froth were observed in the 9:1 and 7:3 mixtures of LSL-ASL as with LSL alone. Hence, ASL is unable to hinder the formation of the non-floatable hydrophobic flocs.

In contrast, in the LSL flotation of hematite, the froth is rich and there are no large agglomerates (Figure S7c).

Thus, the reason for the low floatability of $CeO_2^{(red)}$ with LSL is the formation of hydrophobic agglomerates which act as a deformer.

S6. Hydrophobicity test using high speed camera

For the hydrophobicity test of CeO_2^{red} particles with adsorbed ligands, we used a high-speed camera (see methods). Figure S8 shows the set-up used for the experiment, and example of particles bed deposited on the glass slide. The video of the experiment is available on link [https://youtu.be/SjFNEvwH7-Y]

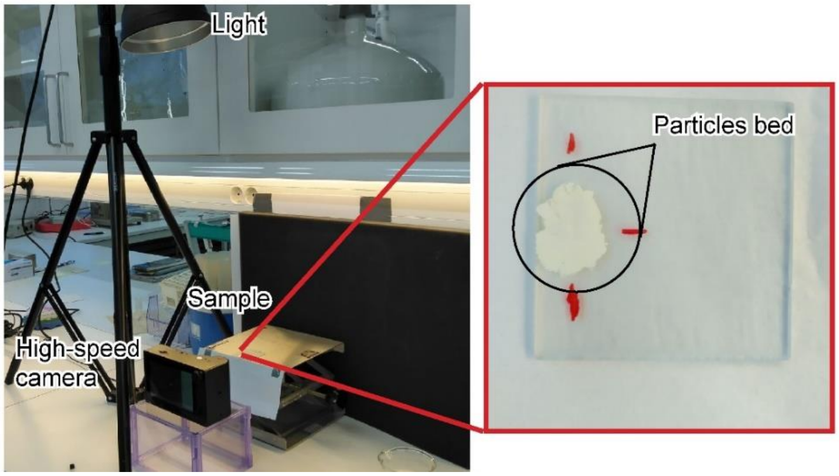

Figure S8 The set-up with high-speed camera and example of $CeO_2^{(red)}$ sample used for hydrophobicity test.

S7. Effect of oxidation of $CeO_2^{(red)}$ on its flotation with ASL and LSL

The treatment of reduced CeO_2 with H_2O_2 is known to partially oxidize the surface Ce^{III} cations to Ce^{IV}.[15, 16] Hence, to oxidized $CeO_2^{(red)}$, we conditioned 10 g of these particles in 30 mL of 30 % H_2O_2 on a shaking table (150 rpm) for 1 h. The dispersion was filtered and dried in a vacuum. XPS spectra confirm that the surface of H_2O_2-treated $CeO_2^{(red)}$ has a lower concentration of Ce^{III} cations than the initial one (Figure S9, see *section 3.1.3* for the peak interpretation).

The H_2O_2-treated $CeO_2^{(red)}$ has a dark yellow color (Figure S9c). Previous studies have attributed this color to the formation of a highly hydrated peroxo-cerium product ($Ce(O_2)(OH)_2/Ce(OH)_4$, which is can oxidize adsorbed organic molecules[17, 18].

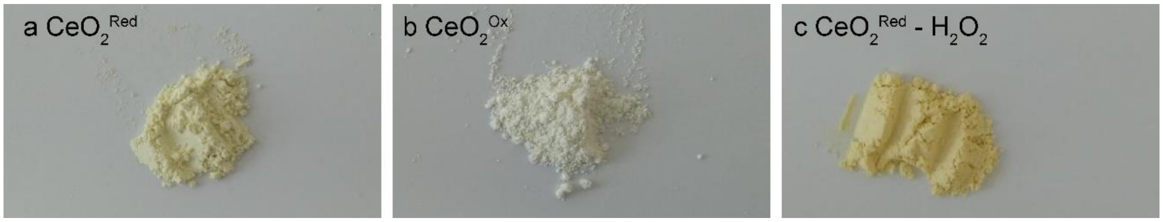

Figure S9 Photos of (a) $CeO_2^{(red)}$, (b) $CeO_2^{(ox)}$, and (c) $CeO_2^{(red)}$ after H_2O_2 treatment

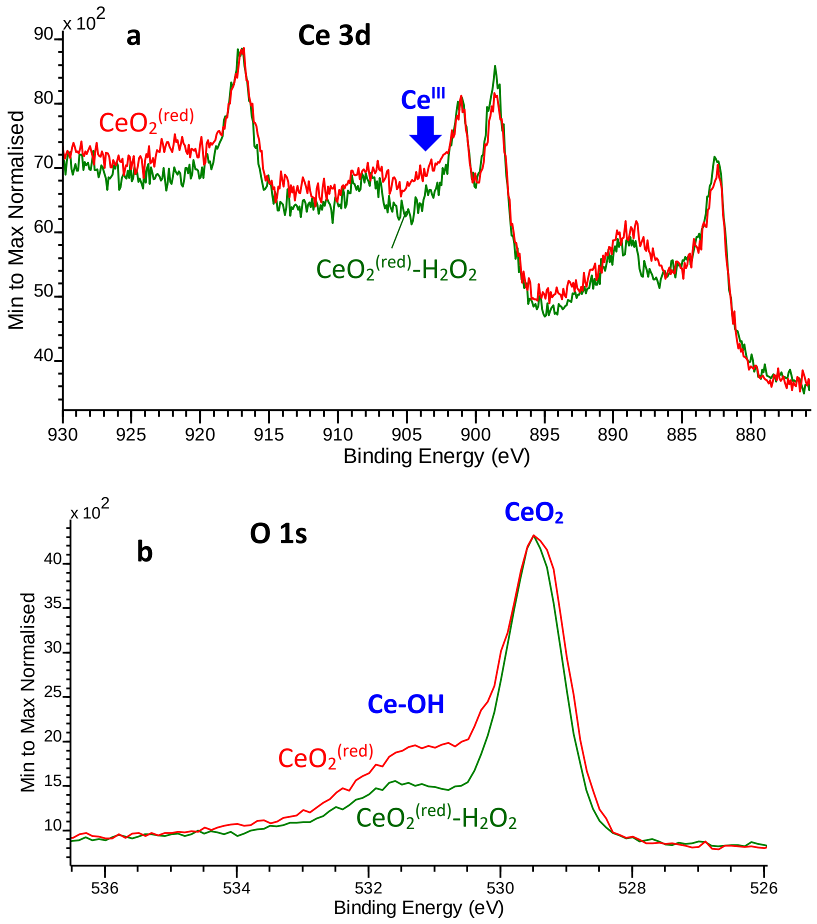

Figure S10 (a) Ce 3d (b) O 1s XPS spectra of (red line) $CeO_2^{(red)}$ and (green line) H_2O_2-oxidized $CeO_2^{(red)}$. As shown in Table S1, the oxidation of $CeO_2^{(red)}$ with H_2O_2 improves the ceria recovery with LSL, which is accompanied by improvement of the froth stability (Figure S11). At the same time, the oxidation slightly suppressed the ceria recovery with ASL. Both these trends reproduce with the trends observed for $CeO_2^{(ox)}$ vs. $CeO_2^{(red)}$. This result rules out the difference in the particles morphology as the main reason for the difference in the floatability of $CeO_2^{(red)}$ and $CeO_2^{(ox)}$. In addition, it further supports the notion that the oxidation state strongly affects the affinity of ceria to O-donor collectors.

Table S2. Flotation of initial and H_2O_2-oxidized $CeO_2^{(red)}$ with ASL and LSL at pH 4

**Biosurfactant and its concentration**	**Recovery of $CeO_2^{(red)}$, %**	**Recovery of $CeO_2^{(red)}$ -H_2O_2, %**
LSL 50 µM	0	13
LSL 100 µM	0	37
ASL 50 µM	70	60
ASL 100 µM	80	72

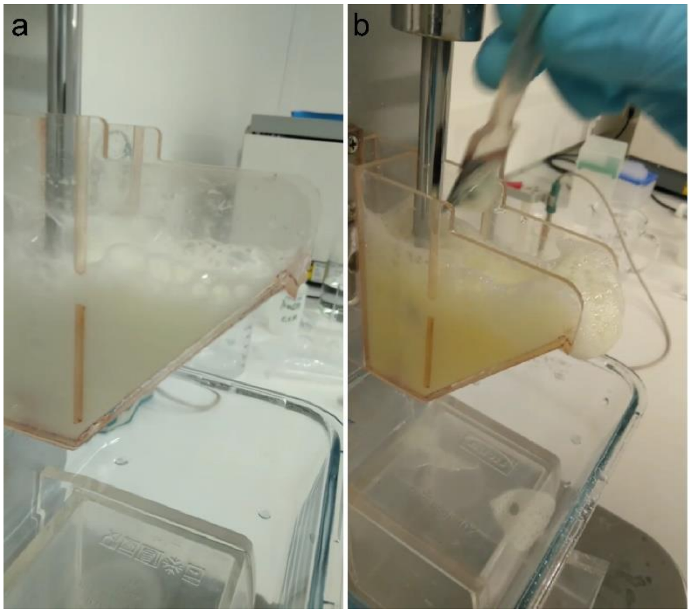

Figure S11 Flotation of (a) $CeO_2^{(red)}$ and (b) H_2O_2-oxidized $CeO_2^{(red)}$. Video link [https://www.youtube.com/watch?v=vUe3lbBHBnc]

S8. XPS of initial and acid-washed hematite

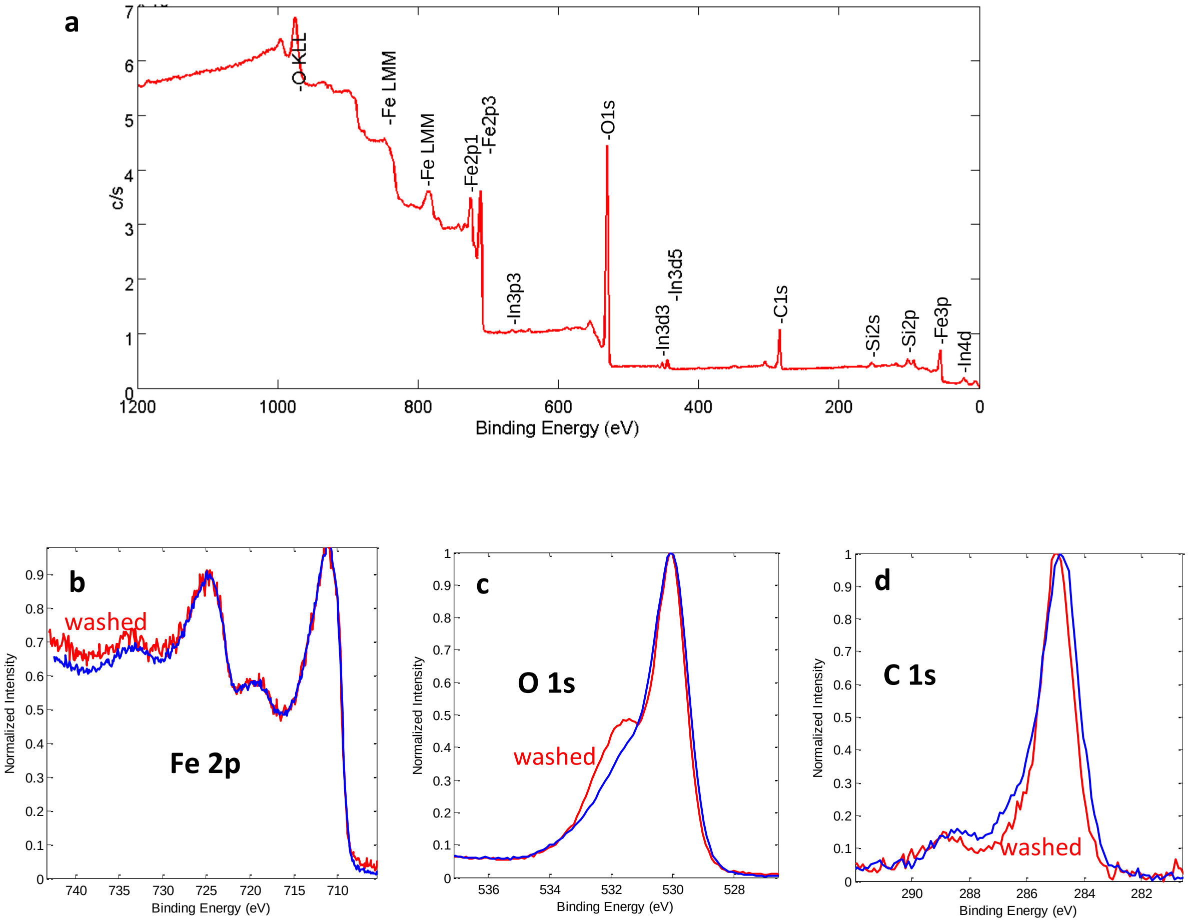

Figure S12 XPS spectra of ultrafine hematite particles: (a) survey spectrum of the initial particles; (b) Fe 2p, (c) O 1s, (d) C1s spectra of (blue) initial and (red) washed overnight in 0.001 M HNO_3. The binding energy was calibrated by aligning the O 1s peak of the hematite lattice at 530.1 eV [19].

The survey spectra of hematite particles (Figure S12a) reveal only traces of Si. The additional In peaks are due to the In support of the particles. The O 1s spectra (Figure S12c) show that the particles washed in a 0.001 M HNO_3 solution overnight are characterized by a higher intensity of the O 1s peak at 531.5 eV which is due to surface hydroxyls.[19] It follows that the washed hematite is more hydrated compared to the initial one.

References

[1] V. Slabov, G. Jain, E. Larsen, H.R. Kota, I. Chernyshova, Eco-Friendly Collectors for Flotation of Fine Hematite and Malachite Particles, Mining, Metallurgy & Exploration, 40 (2023) 475-492.
[2] R.T. Otto, H.J. Daniel, G. Pekin, K. Müller-Decker, G. Fürstenberger, M. Reuss, C. Syldatk, Production of sophorolipids from whey, Applied Microbiology and Biotechnology, 52 (1999) 495-501.
[3] S.J.J. Fleurackers, On the use of waste frying oil in the synthesis of sophorolipids, Eur. J. Lipid Sci. Technol., 108 (2006) 5-12.
[4] D. Möbius, R. Miller, V.B. Fainerman, Surfactants: chemistry, interfacial properties, applications, Elsevier2001.
[5] R.W.G. Wyckoff, Crystal Structure New York 1963.
[6] K. Zhou, X. Wang, X. Sun, Q. Peng, Y. Li, Enhanced catalytic activity of ceria nanorods from well-defined reactive crystal planes, Journal of Catalysis, 229 (2005) 206-212.
[7] C. Yang, X. Yu, S. Heißler, P.G. Weidler, A. Nefedov, Y. Wang, C. Wöll, T. Kropp, J. Paier, J. Sauer, O2 Activation on Ceria Catalysts—The Importance of Substrate Crystallographic Orientation, Angew. Chem. Int. Ed., 56 (2017) 16399-16404.
[8] E. Paparazzo, Use and mis-use of x-ray photoemission spectroscopy Ce3d spectra of Ce2O3 and CeO2, J. Phys.: Condens. Matter, 30 (2018) 343003.
[9] M.M. Khan, S.A. Ansari, D. Pradhan, D.H. Han, J. Lee, M.H. Cho, Defect-Induced Band Gap Narrowed CeO2 Nanostructures for Visible Light Activities, Ind. Eng. Chem. Res., 53 (2014) 9754-9763.
[10] V. Matolín, I. Matolínová, F. Dvořák, V. Johánek, J. Mysliveček, K.C. Prince, T. Skála, O. Stetsovych, N. Tsud, M. Václavů, B. Šmíd, Water interaction with CeO2(1 1 1)/Cu(1 1 1) model catalyst surface, Catalysis Today, 181 (2012) 124-132.
[11] N. Bosio, A. Schaefer, H. Grönbeck, Can oxygen vacancies in ceria surfaces be measured by O1s photoemission spectroscopy?, J. Phys.: Condens. Matter, 34 (2022) 174004.
[12] J.F. Moulder, W.F. Stickle, P.E. Sobol, K.D. Bomben, Handbook of X Ray Photoelectron Spectroscopy: A Reference Book of Standard Spectra for Identification and Interpretation of XPS Data, Perkin-Elmer Corporation, Eden Prairie, MN, 1992.
[13] J.E. Spanier, R.D. Robinson, F. Zhang, S.-W. Chan, I.P. Herman, Size-dependent properties ofCeO2−ynanoparticles as studied by Raman scattering, Physical Review B, 64 (2001).
[14] M. Baalousha, Y. Ju-Nam, P.A. Cole, J.A. Hriljac, I.P. Jones, C.R. Tyler, V. Stone, T.F. Fernandes, M.A. Jepson, J.R. Lead, Characterization of cerium oxide nanoparticles-Part 2: Nonsize measurements, Environ. Toxicol. Chem., 31 (2012) 994-1003.
[15] E.G. Heckert, A.S. Karakoti, S. Seal, W.T. Self, The role of cerium redox state in the SOD mimetic activity of nanoceria, Biomaterials, 29 (2008) 2705-2709.
[16] P. Ji, L. Wang, F. Chen, J. Zhang, Ce3+-Centric Organic Pollutant Elimination by CeO2 in the Presence of H2O2, ChemCatChem, 2 (2010) 1552-1554.
[17] C.J. Neal, T.S. Sakthivel, Y. Fu, S. Seal, Aging of Nanoscale Cerium Oxide in a Peroxide Environment: Its Influence on the Redox, Surface, and Dispersion Character, The Journal of Physical Chemistry C, 125 (2021) 27323-27334.
[18] F.H. Scholes, C. Soste, A.E. Hughes, S.G. Hardin, P.R. Curtis, The role of hydrogen peroxide in the deposition of cerium-based conversion coatings, Appl. Surf. Sci., 253 (2006) 1770-1780.
[19] I.V. Chernyshova, S. Ponnurangam, P. Somasundaran, Adsorption of Fatty Acids on Iron (Hydr)oxides from Aqueous Solutions, Langmuir, 27 (2011) 10007-10018.
